# Excitatory neurons in the lateral parabrachial nucleus mediate the interruptive effect of inflammatory pain on a sustained attention task

**DOI:** 10.1186/s12967-023-04583-9

**Published:** 2023-12-10

**Authors:** Huan-Yu Zheng, Yu-Meng Chen, Yao Xu, Cheng Cen, Yun Wang

**Affiliations:** 1 Neuroscience Research Institute and Department of Neurobiology, School of Basic Medical Sciences, Key Laboratory for Neuroscience, Ministry of Education/National Health Commission and State Key Laboratory of Natural and Biomimetic Drugs, Peking University, Beijing, 100191 China; 2 PKU-IDG/McGovern Institute for Brain Research, Peking University, Beijing, 100871 China

**Keywords:** Acute pain, Ascending pain pathways, Attentional deficits, Chronic pain, Lateral parabrachial nucleus, Sustained attention

## Abstract

**Background:**

Attentional deficits are among the most common pain-induced cognitive disorders. Pain disrupts attention and may excessively occupy attentional resources in pathological states, leading to daily function impairment and increased disability. However, the neural circuit mechanisms by which pain disrupts attention are incompletely understood.

**Methods:**

We used a three-choice serial reaction time task (3CSRTT) to construct a sustained-attention task model in male C57BL/6J mice. Formalin or complete Freund's adjuvant was injected into a paw to establish an inflammatory pain model. We measured changes in 3CSRTT performance in the two inflammatory pain models, and investigated the neural circuit mechanisms of pain-induced attentional deficits.

**Results:**

Acute inflammatory pain impaired 3CSRTT performance, while chronic inflammatory pain had no effect. Either inhibition of the ascending pain pathway by blockade of the conduction of nociceptive signals in the sciatic nerve using the local anesthetic lidocaine or chemogenetic inhibition of Ca^2+^/calmodulin-dependent protein kinase IIα (CaMKIIα) neurons in the lateral parabrachial nucleus (LPBN) attenuated the acute inflammatory pain-induced impairment of 3CSRTT performance, while chemogenetic activation of CaMKIIα neurons in the LPBN disrupted the 3CSRTT. Furthermore, the activity of CaMKIIα neurons in the LPBN was significantly lower on Day 2 after complete Freund's adjuvant injection than on the day of injection, which correlated with the recovery of 3CSRTT performance during chronic inflammatory pain.

**Conclusions:**

Activation of excitatory neurons in the LPBN is a mechanism by which acute inflammatory pain disrupts sustained attention. This finding has implications for the treatment of pain and its cognitive comorbidities.

**Supplementary Information:**

The online version contains supplementary material available at 10.1186/s12967-023-04583-9.

## Background

Pain is a signal warning of actual or potential threats and demands high attentional resources [1]. Due to the limited attentional resources of individuals, pain may disrupt essential attention-demanding tasks, leading to daily functional impairment and increased disability [2, 3]. Attentional deficits are among the most common pain-induced cognitive disorders [4, 5]. Clinical studies have found that both acute and chronic pain affect attentional performance. Experimentally-induced acute pain draws attention away from assigned tasks, and attentional deficits have been found in several chronic pain disorders, and clinical studies have shown an increased incidence of patient-reported attention deficits with chronic pain [6–10]. Similarly, in animal experiments, both acute and chronic pain have been observed to interrupt attention-demanding tasks [11–14]. However, the neural circuit mechanisms by which pain disrupts attention remain inadequately understood.

Functional magnetic resonance imaging studies showed that acute pain could modulate a frontoparietal attention network mobilized by salience detection processes [15], providing evidence for the allocation of attentional resources to pain. Other investigators identified alterations in brain regions that jointly regulate pain and attention in chronic pain; these include the anterior cingulate cortex, insula, and amygdala [16–18]. The overlap between the ascending pain pathways and the neural circuits regulating attention involves mechanisms through which pain disrupts attention.

The lateral parabrachial nucleus (LPBN) is a crucial component of the ascending pain pathways [19]. Excitatory neurons in the LPBN are hyperactivated in several rodent acute and chronic pain models [20–22]. Recent animal studies revealed that the LPBN alerts to threatening stimuli, including painful and non-painful noxious stimuli, inducing negative emotions, coping behaviors, and defensive behaviors [23, 24]. These findings suggest that the LPBN detects the salience of stimuli and attracts attentional resources to respond to threats. Moreover, several brain regions receiving projections from the LPBN, including the central amygdala, the bed nucleus stria terminalis, and the mediodorsal thalamus, are directly or indirectly involved in attentional processing [25–28]. These findings suggest that the increased activity of excitatory neurons in the LPBN participates in the interruptive effect of pain on attention.

In the present study, we used a three-choice serial reaction time task (3CSRTT) to construct a sustained-attention task model in mice. We found that acute inflammatory pain induced by intraplantar injection of formalin or complete Freund's adjuvant (CFA) impaired 3CSRTT performance, while chronic inflammatory pain induced by CFA injection exerted no effect. We also found that activation of excitatory neurons in the LPBN was one of the critical mechanisms by which acute inflammatory pain impairs sustained attention performance. Finally, activation of excitatory neurons in the LPBN was significantly lower in the chronic inflammatory pain state than in the acute inflammatory pain state, which may account for the absence of significant impairment in 3CSRTT performance in the chronic inflammatory pain state.

## Methods

### Animals

Experiments were performed in male C57BL/6 mice (25–30 g, supplied by the Animal Center of Peking University) that were housed in a 12/12-h light/dark cycle and provided food and water ad libitum. A total number of 297 mice were recruited for this study. During 3CSRTT, mice were maintained at 85–90% of their respective baseline free feeding weights. All experimental procedures complied with the guidelines of the Animal Care and Use Committee of Peking University.

### Stereotactic surgery

Mice were anesthetized with pentobarbital (0.05%, 100 mg/kg i.p.), and the head was fixed onto a mouse stereotactic apparatus (Cat#68,030, RWD Life Science, China). Based on the Allen Mouse Brain Atlas (Paxinos & Franklin, 2001) and previous histological verifications, the stereotaxic coordinates for injections/implantations into the LPBN were as follows: AP − 5.20 mm, ML ± 1.30 mm, DV − 3.90 mm. Virus was injected with a microlitre syringe (Cat# 80,135, Hamilton, Switzerland) at a flow rate of 30 nL/min, the syringe was withdrawn 5 min after the end of the injection. Mice were isolated on a heating pad until fully-awake and were returned to their home cages. Penicillin was subcutaneously injected for 3 consecutive days post-surgery to prevent infection. To ensure the full expression of the virus and full recovery of the mice, behavioral experiments were performed 2 weeks after surgery. The expression and site of the virus were histologically verified following behavioral experiments, and any individual mice with inadequate expression or unverifiable injection sites were excluded.

For chemogenetic experiments, 60–80 nL of adeno-associated virus (AAV) 2/9-CaMKIIα-hM4D(Gi)-mCherry-WPRE-hGH (BrainVTA Technology, China) or AAV2/9-CaMKIIα-hM3D(Gq)-mCherry-WPRE-hGH (BrainVTA Technology, China) was injected bilaterally while AAV2/9-CaMKIIα-mCherry-WPRE-hGH (OBiO Technology, China) was injected as a control.

For in vivo imaging experiments, 150 nL of AAV2/9-CaMKIIα-GCaMP6s -WPRE-hGH (BrainVTA Technology, China) was injected unilaterally while AAV2/9-CaMKIIα-EGFP -WPRE-hGH (BrainVTA Technology, China) was injected as control. After virus injection, a single fiber implant (200 μm, diameter = 1.25 mm, NA = 0.37) was placed 0.2 mm above the injection site, and was secured to the skull with two screws and dental cement.

### Behavioral assays

All behavior tests were conducted during the light cycle (7:00–19:00). Mice were habituated to investigator handling for 5 min per day on 3 consecutive days. One hour prior to each test, mice were transported to the behavior room to habituate. Data were analysed by researchers blinded to the experimental groups.

For chemogenetic experiments, either clozapine N-oxide (CNO, diluted with saline, Cat# S6887, Selleck, China) or saline was injected intraperitoneally (2.5 mg/kg CNO) 30 min before each behavior test.

#### 3CSRTT

The 3CSRTT protocol was modified as described [29]. Mice were required to direct their attention to the location of a visual stimuli to obtain a food reward in an operant chamber. The operant chamber (Harvard Apparatus, USA) had a house light on the top and nine holes with internal light-emitting diodes (LED lights) on the front wall. Three of these holes were used to present the visual stimuli (cues), and each hole was equipped with an infrared sensor to detect nose pokes by the mouse into the hole. There was also a reward magazine on the back wall that dispensed food pellets and was equipped with an infrared sensor to detect retrieval of the food rewards by mice. During the whole task, mice were maintained at 85%-90% of their original body weight to maintain their desire for food rewards. Each mouse was trained in the same operant chamber every time. The training protocol included pretraining and four training stages with gradually increasing difficulty (Additional file 1: Fig. S1).

Before the pre-training stage began, mice were firstly allowed to habituate the operant chamber for one day, food pellets were placed in various holes and the reward magazine. For the pre-training stage, mice were allowed to explore the operant chamber and learn to poke the hole with nose when the LED light of the hole was on. During pre-training, one of three LED lights was illuminated for 15 min, once the nose poke was detected, which represented a correct response, mice received a food pellet from the reward magazine. The training stage began when the number of correct responses exceeded 15 times within 15 min in the pre-training test (Additional file 1: Fig. S1D).

In the training test, mice were trained in four consecutive training stages, each with a specific criterion. The criteria of each stage needed to be met for two consecutive days before the mice could progress to the next stage. During the training test, one of three LED lights was illuminated within a limited duration (gradually from 30 s down to 2 s as training progressed through different stages) as a visual cue, followed by a limited hold time (gradually from 10 s down to 5 s) for mice to make a decision. The timepoint from trial start to cue onset was defined as the inter-trial interval. A nose-poke into the illuminated hole within the limited hold period was counted as a correct response, and mice would receive a food reward. Mice might also exhibit three types of error responses: an incorrect response (a nose poke into a non-illuminated, or LED-off, hole), an omission response (a failure to respond within the limited hold period), or a premature response (a nose poke during the inter-trial interval). These error responses triggered a 2-s timeout period as a punishment (with house light on). The next trial started automatically 12 s after a correct response or a timeout period. The number of correct responses and error responses as well as the correct response latency (the time from cue onset to the correct response) and reward latency (the reaction time to collect the food reward in a correct trial) were recorded. A training session ended either after completing 50 trials or continuing for 30 min.

In the testing stage, sessions lasted for 50 trials or 60 min according to experimental demands. The cue duration was reduced to 2 s. ITIs of 3, 4, or 5 s were presented pseudo-randomly on a trial-to-trial basis to prevent self-pacing strategies for the prediction of cue onset (Additional file 1: Fig. S1E–I).

The raw data of the 3CSRTT were recorded by PACKWIN V2.0 software (Harvard Apparatus, USA), and the calculation methods for the main parameters were as follows:

1. Correct response rate = number of correct responses/total number of trials * 100%
2. Incorrect response rate = number of incorrect responses/total number of trials * 100%
3. Omission response rate = number of omission responses/total number of trials * 100%
4. Premature response rate = number of premature responses/total number of trials * 100%
5. The correct response latency and reward latency were directly recorded by the software (average data for each mouse).

#### Formalin test

Mice were subcutaneously injected with 20 μL of 2% formalin into a hind paw; control mice were injected with an equal amount of 0.9% saline, and the subsequent spontaneous nociceptive behaviour (licking the injected paw) was recorded for 60 min using an infrared camera.

#### Chronic inflammatory pain model

To induce chronic inflammatory pain, 30 μL of complete Freund's adjuvant (CFA; Cat# F5881, Sigma‒Aldrich, USA) was subcutaneously injected into a hind paw. Control mice were injected with an equal amount of 0.9% saline.

#### Mouse sciatic nerve blockade model

The experimental method was mainly referred to [30, 31]. Before the experiment started, the mice were taught to walk normally on a wire grid mesh with mesh holes approximately 5 mm in diameter. All mice were able to use all four limbs to hang off and crawl when placing them on inverted mesh before treatments. Mice were slightly restrained and the injection was administered into the sciatic nerve area inside the popliteal fossa of the left hind limb, with a constant volume of 50 μL for all drugs. The primary endpoint was the time to loss of ability to hang on to the inverted mesh with the injected hind limb, which was tested at 1, 5, 10, 20, 30, 40, 50 and 60 min after injection.

#### Hargreaves test

The Hargreaves test was used to assess paw withdrawal latencies in response to radiant heat. Prior to the test, mice were placed inside a transparent organic glass cover (18 cm × 8 cm × 8 cm) on an elevated glass platform and were allowed to adapt for 20 min until their grooming and exploratory activities subsided. The radiant heat source was adjusted to a range of 10–15 s for mice as the baseline latencies. During the test, the radiant heat source under the glass platform was adjusted to point at the plantar surface of the mouse hind paw. When the mice quickly removed their hind paw from the heating spot, the radiant heat source was immediately turned off, and the time interval of the on–off switch was recorded. If there was no positive response after 30 s of heating, then the radiant heat source was switched off immediately to prevent tissue damage. Each mouse underwent three repeated tests, and the average was taken; there was a minimum 5-min interval between tests.

#### Spatial working memory test

A Y-maze (Cat# 63,006, RWD Life Science, China) was used to measure spatial working memory. The apparatus consisted of three enclosed arms with dimensions of 50 cm × 8 cm × 15 cm (length × width × height) arranged at a 120° angle. Each arm had a movable partition on the centre side, and different geometric shaped stickers were pasted on the inside wall of each arm as visual cues. During the test, each mouse was randomly placed in one of the three arms of the Y-maze and was allowed to explore freely for 5 min while spontaneous alternation behaviours were recorded and analysed. A consecutive entry into three different arms was counted as one alternation event. Spontaneous alternation (%) = [(number of alternations)/(total number of arm entries−2)] × 100.

#### Short-term spatial memory test

The Y-maze (Cat# 63,006, RWD Life Science, China) was used to measure short-term spatial memory. The test consisted of a learning phase, a retention phase and a testing phase. In the learning phase, each mouse was placed in the starting arm with one of two other arms closed and was allowed to freely explore for 5 min. The retention phase lasted for 2 min, during which mice were moved back to their home cages. In the testing phase, the previously closed arm (designated herein as the “New Arm”) was open, and each mouse was placed in the starting arm and allowed to freely explore for 5 min. The percentage of time spent in the new arm was recorded and analysed.

#### Conditioned place aversion (CPA) test

The experimental method mainly referred to [24]. The CPA apparatus (Cat#63,020, RWD Life Science, China) (56 cm × 25 cm × 25 cm) consisted of two chambers distinguished by visual cues on the wall and a central connecting corridor (8 cm × 5 cm). On Day 1 (pre-conditioning), mice were placed in the central corridor of the apparatus and were allowed to freely explore for 20 min, while the time spent in each area was recorded and analysed.

On Days 2 and 3 (conditioning), mice were placed in one chamber (conditioned chamber) of the apparatus for 20 min each morning and afternoon. For the hM3Dq-induced CPA paradigm, mice received intraperitoneal injection (i.p.) 30 min before conditioning started: 0.09% saline i.p. in the morning, followed by clozapine N-oxide (CNO, 2.5 mg/kg) i.p. in the afternoon. For the formalin-induced CPA paradigm, mice did not receive any treatment in the morning but received 10 μL subcutaneous injection of 2% formalin into either the contralateral or ipsilateral hind paw in the afternoon. For the hM4Di rescue paradigm, mice received CNO (2.5 mg/kg) i.p. 30 min prior to formalin administration.

On Day 4 (post-conditioning), mice were placed in the central corridor of the apparatus and were allowed to freely explore for 20 min, while the time spent in each area was recorded and analysed.

### Brain slice preparation and immunohistochemistry

Mice were anesthetized by pentobarbital (0.05%, 100 mg/kg i.p.) and underwent transcardial perfusion with phosphate-buffered saline (PBS) followed by 4% paraformaldehyde (PFA). Brains were extracted and post-fixed with 4% PFA overnight, followed by dehydration with 20% sucrose in PBS for 24 h and then 30% sucrose in PBS for 24 h. Each brain was embedded in Optimum Cutting Temperature (OCT, Tissue Tek) in a 1.5 cm × 1.5 cm × 1.5 cm capacity folded with aluminum foil paper, and sectioned into 30 μm coronal slices using a cryostat (CM3050, Leica, GER). Slices were mounted on a slide (2–6 slices per slide) and 37 °C dried overnight, and finally were covered with mounting medium (antifading, with DAPI, Solarbio Life Science, China) under glass coverslips. Images were observed using confocal microscope (TCS-SP8 DIVE, Leica, GER).

For immunohistochemistry, brain slices on the slides were placed at room temperature for 10 min before washing with PBS 3 times (5 min each) and were incubated with blocking buffer (3% bovine serum albumin and 0.3% Triton X-100 dissolved in PBS) for 1 h at room temperature. Brain slices were incubated with primary antibody in antibody dilution buffer (1% BSA and 0.1% Triton X-100 dissolved in PBS) for 24 h at 4 °C and were washed 3 times (5 min each) with PBS before overnight incubation with secondary antibody. After 3 washes (5 min each) with PBS, the slides were dried and then covered with mounting medium under glass coverslips.

The antibodies used for staining were as follows: rabbit anti-cFos (1:500, RRID: AB_2247211, Cell Signaling Technology, USA), anti-rabbit Alexa-488 (1:1000, Cat# A11034, Invitrogen, USA), mouse anti-CaMKIIα (1:1000, RRID: AB_2721906, Cell Signaling Technology, USA) and anti-mouse Alexa-594 (1:1000, RRID: AB_141633, Invitrogen, USA).

### Calcium imaging in vivo

Stereotactic injection of AAV-GCaMP6s and fibre implantation in LPBN were conducted. After 2 weeks of recovery, mice were habituated for 3 consecutive days with the optical fibre connected. Before each recording, mice with the optical fibre connected were allowed to adapt for 30 min. Recording during behaviour experiments was conducted using a monochromatic single channel optical fibre recording system (Thinker Tech, China), and the behaviour of mice was collected using an infrared camera. Calcium signal acquisition and video recordings were manually synchronized.

For recording during the formalin test, 5-min baseline calcium signals were collected as controls before formalin injection into the contralateral hind paw, followed by calcium imaging and behavioural recording for 60 min. For recording under CFA-induced pain, 10-min baseline calcium signals were collected as controls before 30-min calcium imaging, and behavioural recordings were collected as event-related periods on the day before injection (-1 d), the day of injection (0 d), and Day 2 after injection (2 d).

ΔF/F refers to the relative change in the fluorescent signal, which was calculated as follows: ΔF/F = (F_T_−F_0_)/(F_0_−F_offset_), with F_T_ representing the mean value of the fluorescent signal during the event-related period; F_0_ representing the mean value of the fluorescent signal during baseline; and F_offset_ representing the background fluorescent signal. The results were analysed using MATLAB 2017a and are presented as a peri-event plot and heatmap.

For recording during the formalin test, the timepoint of the formalin injection was set as the event starting point (zero point), and the calcium signal data from -300 s to 3600 s were selected and analysed in MATLAB. In addition, the mean ΔF/F in phase 1, interphase and phase 2 were calculated. For recording under CFA-induced pain, 10 min after recording was set as the zero point, and the calcium signal data from – 600 s to 1800 s were selected and analysed in MATLAB. The area under the curve during 0–1800 s was calculated using the trapz function.

### Data analysis

Statistical analyses were performed using GraphPad Prism 8 software. All data are presented as the mean ± standard error of the mean (mean ± SEM). To test statistical significance, unpaired t test, paired t test, one-way analysis of variance (ANOVA) followed by the Tukey multiple comparison test, and two-way ANOVA with repeated measures followed multiple comparisons with the Sidak correction were performed when appropriate. A P value less than 0.05 referred to significant differences, which are presented in all figure legends as follows: *p < 0.05; **p < 0.01; ***p < 0.001. All statistical analyses are presented in Additional file 2: Table S1.

## Results

### Formalin-induced acute inflammatory pain impairs 3CSRTT performance

**Fig. 1:**
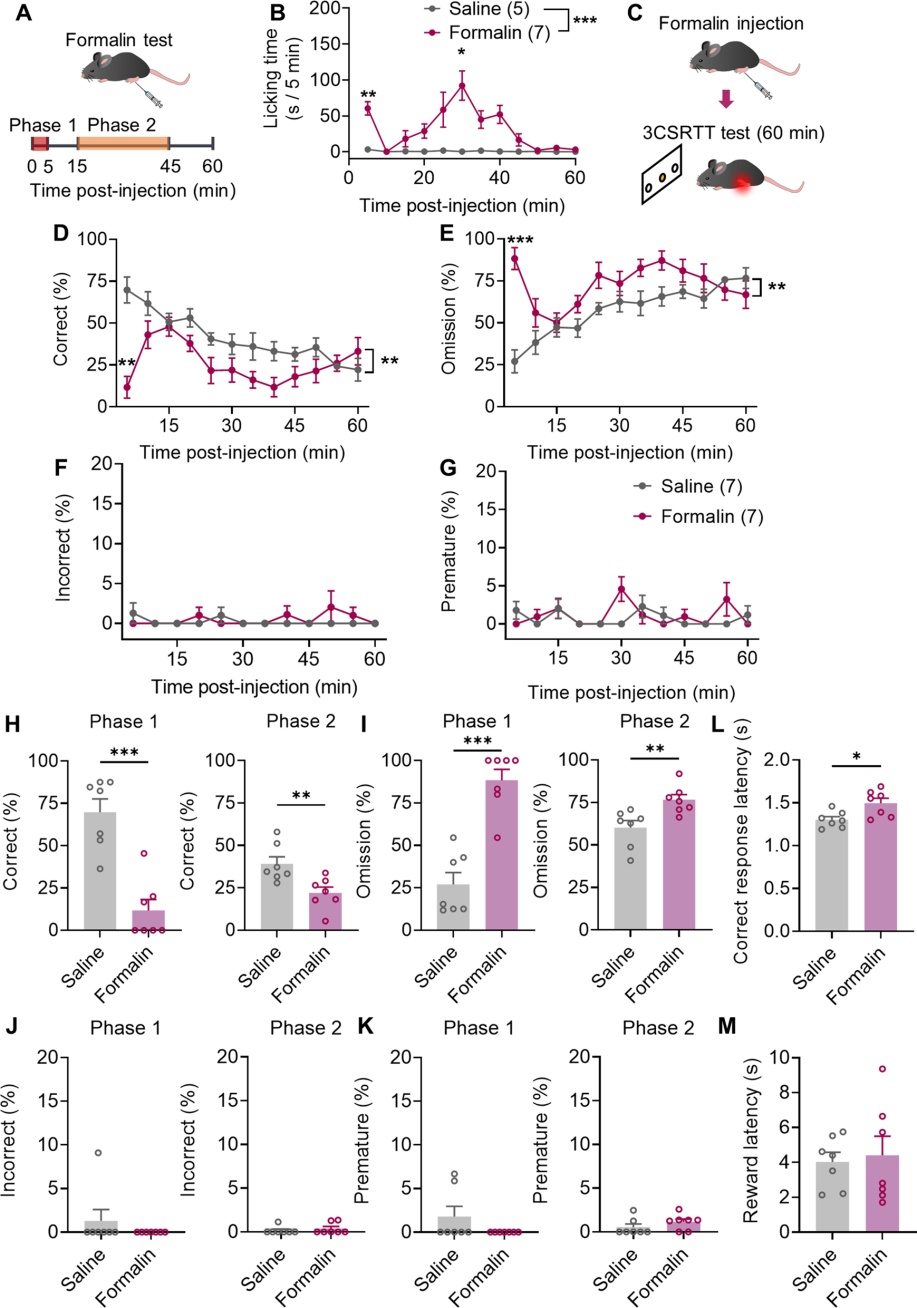
Formalin-induced acute inflammatory pain impairs 3CSRTT performance. **A** Schematic of the formalin test. Paw injection of 2% formalin was administered at 0 min; the time spent licking the paw was measured for 60 min. **B** The time spent licking the paw following saline (n = 5 mice) or formalin (n = 7 mice) injection displayed in 5-min time bins. **C** Experimental design. 3CSRTT was performed immediately after formalin injection for 60 min. **D**–**G** Effects of saline or formalin injection on correct (**D**), omission (**E**), incorrect (**F**), and premature (**G**) responses in the 3CSRTT over the 60-min testing period. Data points are displayed in 5-min time bins. **H**–**K** Effects of saline or formalin injection on correct (**H**), omission (**I**), incorrect (**J**), and premature (**K**) responses in the 3CSRTT in phases 1 and 2. **L**, **M** Effects of saline or formalin injection on correct response latency (**L**) and reward latency (**M**) in the 3CSRTT (n = 7 mice per group). Data are mean ± SEM. *p < .05, **p < .01, ***p < .001. Two-way ANOVA followed by the Sidak’s multiple comparisons test in **B, D**–**G**. Two-tailed unpaired t test in **H**–**M**

We investigated the effect of acute inflammatory pain on attention. A 3CSRTT was used to assess sustained attention in mice. In the task, animals were required to orient to an array of stimulus-presentation holes and to direct their attention to the location of a brief visual stimulus (cue) presented pseudorandomly in one of three presentation holes (Additional file 1: Fig. S1A, B) [29]. The mice were trained using a four-step training schedule defined by specific criteria to learn the task (Additional file 1: Fig. S1C–F). Intraplantar formalin injection was used to induce acute inflammatory pain. Two-percent formalin paw injection induced two distinct phases of pain response characterized by spontaneous paw-licking behavior. The early phase occurred within 5 min (phase 1) following formalin injection, and a late phase (phase 2) lasted from 15 to 45 min (Fig. 1A, B). 3CSRTT was performed immediately after saline or formalin injection for 60 min (Fig. 1C). Intraplantar formalin injection decreased the correct rate and increased the omission rate during phases 1 and 2, while there were no significant differences in interphase and 45–60 min after phase 2 (Fig. 1D, E, H and I). Formalin injection did not affect the rate of incorrect and premature responses (Fig. 1F–K). Formalin injection increased the correct response latency but did not affect reward latency (Fig. 1L, M). These results suggest that formalin-induced acute inflammatory pain disrupts sustained-attention performance in the 3CSRTT, since if increases in omissions are not accompanied by changes in reward latency, the increased omissions may well be due to gross impairments in attention [32].

To investigate whether inflammatory pain induced by formalin injection led to a long-term impairment of 3CSRTT performance, we tested the 3CSRTT performance one day after saline or formalin injection. No significant change was observed between the saline-injected and formalin-injected mice (Additional file 1: Fig. S2). These findings suggest that formalin-induced acute inflammatory pain impaired performance on sustained-attention tasks only in phases 1 and 2.

According to clinical studies, intraplantar injection induces transient pain that interrupts attention within 1500 ms [33]. To investigate whether the transient pain caused by intraplantar injection had effects on the 3CSRTT, we compared the 3CSRTT performance between naive mice and saline-injected mice. No significant change was found between the saline and naive groups in the 3CSRTT (Additional file 1: Fig. S3).

If memory and appetite are affected by pain, their alteration can interfere with attentional performance. To exclude the effect of memory on attentional performance, we investigated the effect of intraplantar formalin injection on spatial working memory and short-term spatial memory using a Y-maze in phases 1 and 2. Formalin-injected mice showed no significant deficit in spatial working memory or short-term spatial memory in the Y-maze tests measured by spontaneous alteration (Additional file 1: Fig. S4A–C) or times in the new arm (Additional file 1: Fig. S4D–F). To exclude the effect of food desire, we measured food intake over 1 h after saline or formalin injection. No significant difference was observed between the saline-injected and formalin-injected mice (Additional file 1: Figs. S5A–C).

### CFA-induced acute inflammatory pain but not chronic inflammatory pain impairs 3CSRTT performance

**Fig. 2:**
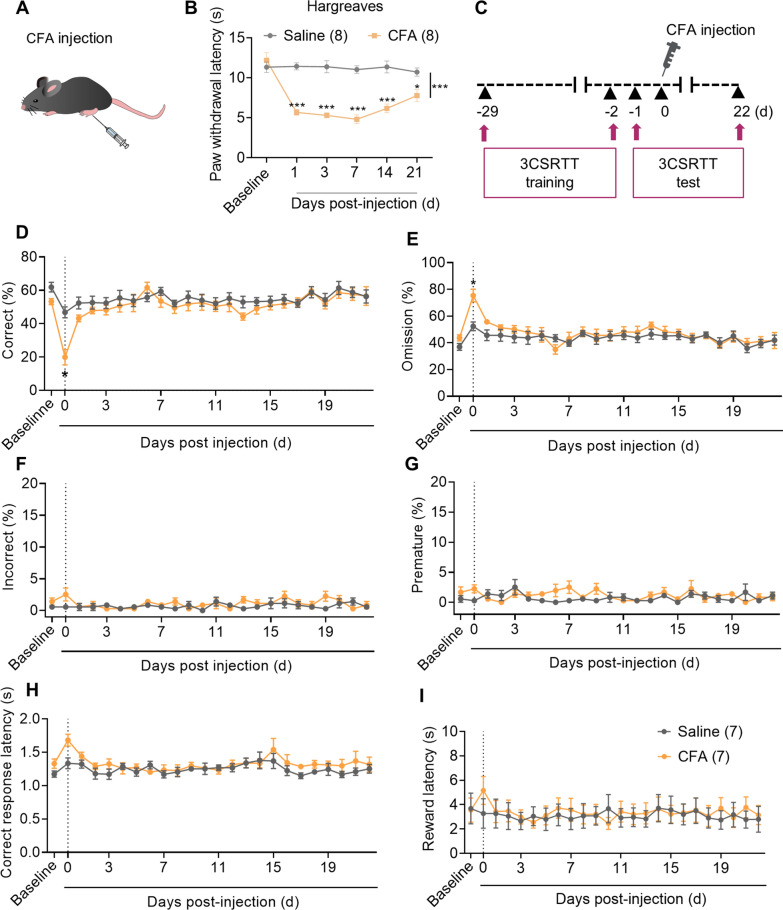
The effect of CFA-induced inflammatory pain on 3CSRTT performance. **A** Schematic of CFA paw injection. **B** Withdrawal latency of the ipsilateral hind paws after saline or CFA injection (n = 8 mice per group). **C** Experimental design. The 3-CSRTT training (50 trials per day) was performed from Day -28 to Day -2 and the 3-CSRTT testing was performed from Day -1 (baseline) to Day 22. CFA was injected on Day 0. **D**–**I** Effects of saline or CFA injection on correct rate (**D**), omission rate (**E**), incorrect rate (**F**), premature rate (**G**), correct response latency (**H**) and reward latency (**I**) in the 3CSRTT. Data are mean ± SEM. *p < 0.05, ***p < 0.001. Two-way ANOVA followed by the Sidak’ s multiple comparisons test

The CFA-induced inflammatory pain model is commonly used because the resulting hyperalgesia can last for a relatively long time (14 to 21 days) (Fig. 2A, B). To investigate the effect of CFA-induced acute and chronic inflammatory pain on sustained attention, we measured changes in 3CSRTT performance in CFA-injected mice (Fig. 2C). CFA-injected mice showed a decreased rate of correct responses and an increased rate of omissions immediately after CFA injection (CFA 0 d) compared with saline-injected mice (Fig. 2D, E and H), while there were no significant differences in the rate of incorrect or premature responses, the correct response latency, or the reward latency (Fig. 2F–I). However, there were no significant differences in 3CSRTT performance between the CFA-injected and saline-injected groups from 1 to 22 days after injection (Fig. 2). These findings suggest that CFA-induced acute inflammatory pain impairs 3CSRTT performance, while CFA-induced chronic inflammatory pain has no effect.

To exclude the effect of memory on attentional performance, we investigated the effect of intraplantar CFA injection on spatial working memory and short-term spatial memory using a Y-maze. CFA-injected mice showed no significant deficit in spatial working memory or short-term spatial memory function in the Y-maze tests measured by spontaneous alternation or times in the new arm (Additional file 1: Fig. S4G, H). Taken together, these findings suggest that acute inflammatory pain but not chronic inflammatory pain impairs performance on the sustained-attention task.

### Blockage of ascending pain pathways attenuates formalin-induced impairment of 3CSRTT performance

**Fig. 3:**
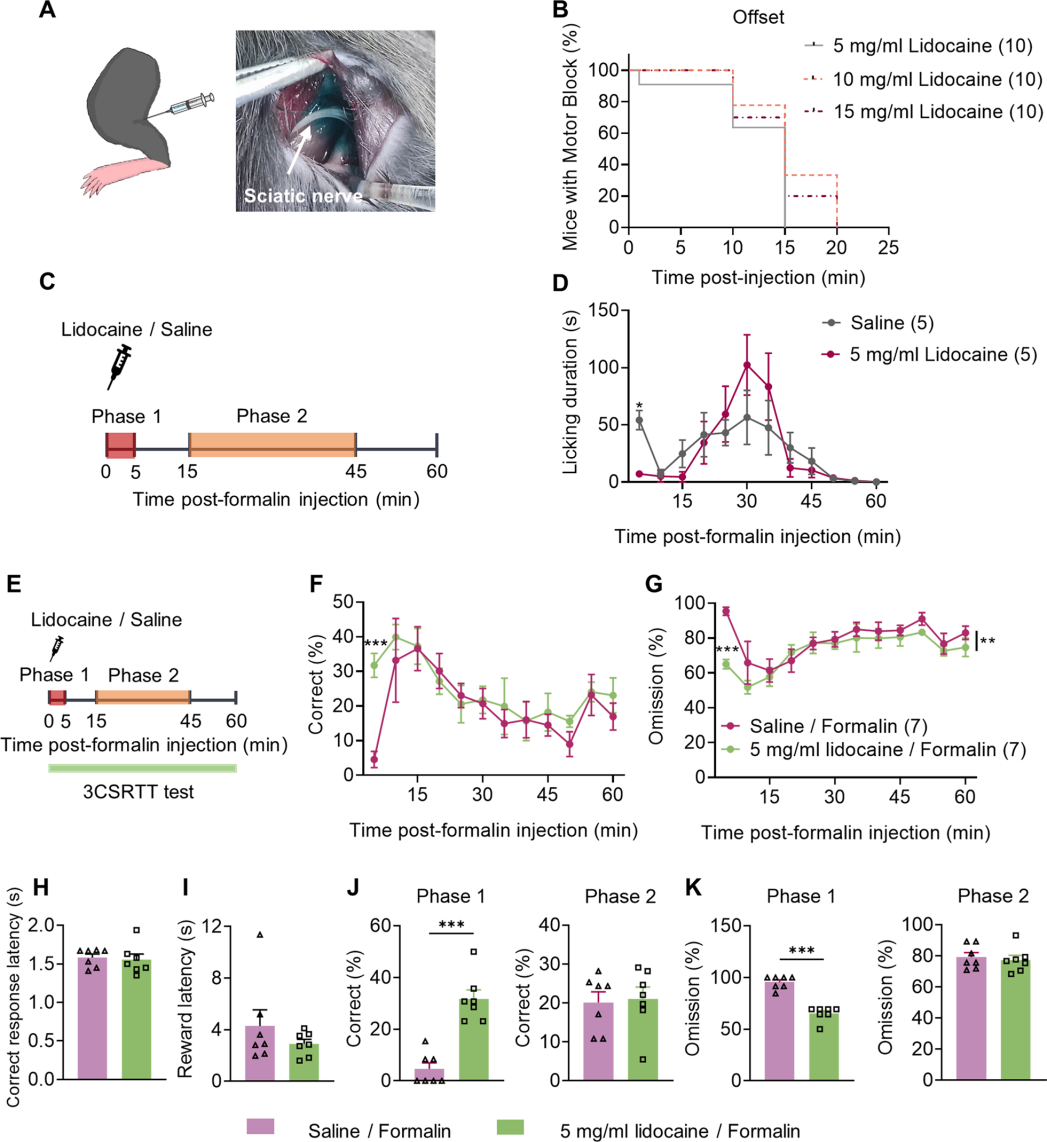
Lidocaine-induced sensory blockade attenuates the impairment of 3CSRTT performance induced by acute inflammatory pain. **A** Schematic of lidocaine injection into the popliteal space and representative images of the sciatic nerve region after injection of 50 μL fast green. **B** Concentration-dependent time-to-event survival curves for offsetting mouse sciatic nerve motor block due to lidocaine injection. The offset time was defined as when the animal regained its ability to use the treated hind limb to hang on to the inverted mesh. (n = 10 per group). **C** Experimental design. Mice were pretreated with either saline or lidocaine in the popliteal space and then injected with 2% formalin. The time spent licking the injected paw was measured for 60 min after formalin injection (n = 5 mice per group). **D** Time spent licking the paw following saline/formalin or lidocaine/formalin injection displayed in 5-min time bins. **E**–**K** The effect of lidocaine-induced sciatic nerve blockade on formalin-induced impairment of 3CSRTT performance. **E** Experimental design. Mice were pretreated with either saline or lidocaine into the popliteal space and then injected with 2% formalin (saline/formalin group or lidocaine/formalin group), and a 60-min 3CSRTT was performed following formalin injection. **F**, **G** Correct (**F**) and omission **(G)** in the 3CSRTT of each group over the 60-min testing period. Data points are displayed in 5-min time bins. **H**, **I** Correct response latency (**H**) and reward latency (**I**) in the 3CSRTT of each group over the 60-min testing period. **J**, **K** Correct (**J**) and omission (**K**) in the 3CSRTT of each group in phases 1 and 2 (n = 7 mice per group). Data are mean ± SEM. *p < 0.05, **p < 0.01, ***p < 0.001. Survival fractions were calculated using the product limit (Kaplan–Meier) method; the survival curves were compared with the log-rank test (**C**). Two-way ANOVA followed by the Sidak’s multiple comparisons test in **D**, **F** and **G** and two-tailed unpaired t test in **H–K**

Clinical studies suggest an overlap between neuro-anatomical cerebral circuits of pain and attention [15], and acute pain may disrupt attention by activating these circuits when activating ascending pain pathways. To determine whether activation of the ascending pain pathway is necessary for the interruptive effect of acute inflammatory pain on attention, we used the lidocaine-induced sciatic nerve blockade model to block the ascending pain pathway [30]. We injected 50 μL of lidocaine into the popliteal space to inhibit voltage-gated Na^+^ channels in the sciatic nerve (Fig. 3A). The effect of lidocaine injection-induced sciatic nerve motor block, which prevented mice from using the injected hind limb to hang on to an inverted mesh, lasted for approximately 10 min; 5, 10, and 15 mg/ml lidocaine showed no significant differences in the offset of the sciatic nerve motor block (Fig. 3B). In the formalin test, injection of 5 mg/ml lidocaine into the popliteal space attenuated paw-licking behavior in phase 1 but did not affect phase 2 because of the limited retention time (Fig. 3C, D). This finding suggests that lidocaine injection blocked the sensory conduction of the sciatic nerve.

To investigate whether sciatic nerve blockade attenuated formalin-induced impairment of 3CSRTT performance, we first assessed the effect of 5 mg/ml lidocaine injection on 3CSRTT under physiological conditions; there was no significant change in 3CSRTT performance in lidocaine-injected mice (Additional file 1: Fig. S6). Then, we assessed the effect of 5 mg/ml lidocaine injection on 3CSRTT in formalin-induced acute inflammatory pain (Fig. 3E). Lidocaine-injected mice showed an increased correct rate and decreased omission rate in phase 1, while there were no significant differences in phase 2 (Fig. 3F, G, J and K). No significant correct response or reward latency change was observed in lidocaine-injected mice. (Fig. 3H, I). However, lidocaine-injected mice showed slightly increased incorrect and premature rates (Additional file 1: Fig. S7). These findings suggest that the activation of ascending pain pathways is necessary for formalin-induced impairment of sustained-attention performance.

### Formalin-induced acute inflammatory pain activates CaMKIIα neurons in the LPBN

**Fig. 4:**
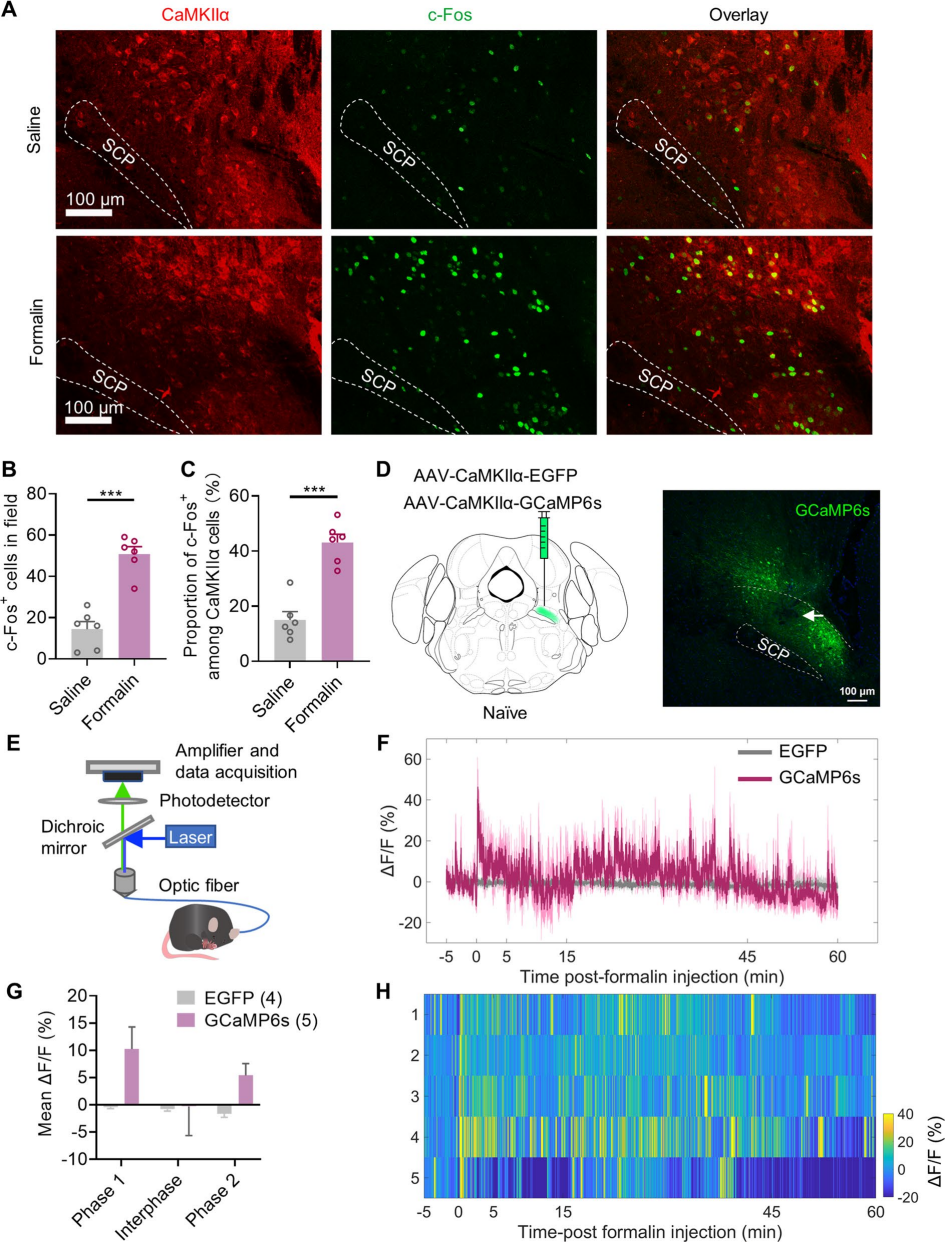
The activity of CaMKIIα neurons in the LPBN is increased after formalin injection. **A-C** Immunofluorescence staining of c-Fos and CaMKIIα in the LPBN after formalin injection. **A** Representative image of co-labeling of CaMKIIα-positive neurons (red) and c-Fos immunoreactivity (green) in the LPBN 1.5 h after saline or formalin injection. **B** Density of c-Fos-positive cells in the LPBN. **C** Proportions of CaMKIIα-positive cells co-expressing c-Fos in the LPBN (n = 6 sections from three mice per group). **D**–**H** In vivo calcium imaging of CaMKIIα neurons in the LPBN after formalin injection. **D** Schematic of unilateral LPBN stereotaxic injection of AAV-CaMKIIα-EGFP or AAV-CaMKIIα-GCaMP6s in naive mice and representative image of expression of the GCaMP6s in the CaMKIIα neurons of LPBN. The arrow indicates the implant location. Scale bar, 100 μm. **E** Schematic of the recording system for the Ca^2+^ signal in the CaMKIIα neurons of LPBN with fiber photometry in naive mice. **F** Average fluorescence signals (ΔF/F) of GCaMP6s (red) and EGFP (grey) recorded from CaMKIIα neurons in the LPBN after formalin injection; baseline was a 5 min period before formalin injection. **G** Mean ΔF/F of GCaMP6s and EGFP fluorescence signals from CaMKIIα neurons in the LPBN in phase 1 (0–5 min), interphase (5–15 min) and phase 2 (15–45 min). **H** Heatmap of ΔF/F for all individual mice with GCaMP6s (n = 5). Data are mean ± SEM. *p < 0.05. Two-tailed unpaired t test in **B**, **C**, and two-way ANOVA followed by the Tukey’s multiple comparisons test in **E**

The LPBN is a crucial component of the ascending pain pathways and may participate in the interruptive effect of pain on attention. In the LPBN, Ca^2+^/calmodulin-dependent protein kinase IIα (CaMKIIα) neurons can represent most of the excitatory neurons [20]. We determined whether CaMKIIα neurons in the LPBN were activated by formalin-induced acute inflammatory pain. Immunostaining of c-Fos at 1.5 h after formalin injection revealed a dramatic increase in the density of c-Fos-positive cells co-expressing CaMKIIα in the LPBN (Fig. 4A, B). Approximately 43% of CaMKIIα neurons were activated (co-localized with c-Fos) in formalin-injected mice compared to 13% in saline-injected mice (Fig. 4C). After formalin injection, we measured the activities of CaMKIIα neurons in the LPBN using in vivo calcium imaging. We injected the AAV2/9-CaMKIIα-GCaMP6s virus or AAV2/9-CaMKIIα-EGFP virus as a control into the LPBN of naive mice (Fig. 4C–E), and fluorescence signals were measured three weeks after injection. Significant Ca^2+^ elevations in CaMKIIα neurons were detected in phases 1 and 2 following formalin injection (Fig. 4F–H). These results suggest that CaMKIIα neurons in the LPBN are involved in acute inflammatory pain and that the activity of CaMKIIα neurons in the LPBN increases after formalin injection.

### Chemogenetic inhibition of CaMKIIα neurons in the LPBN attenuates the impairment of 3CSRTT performance induced by acute inflammatory pain

**Fig. 5:**
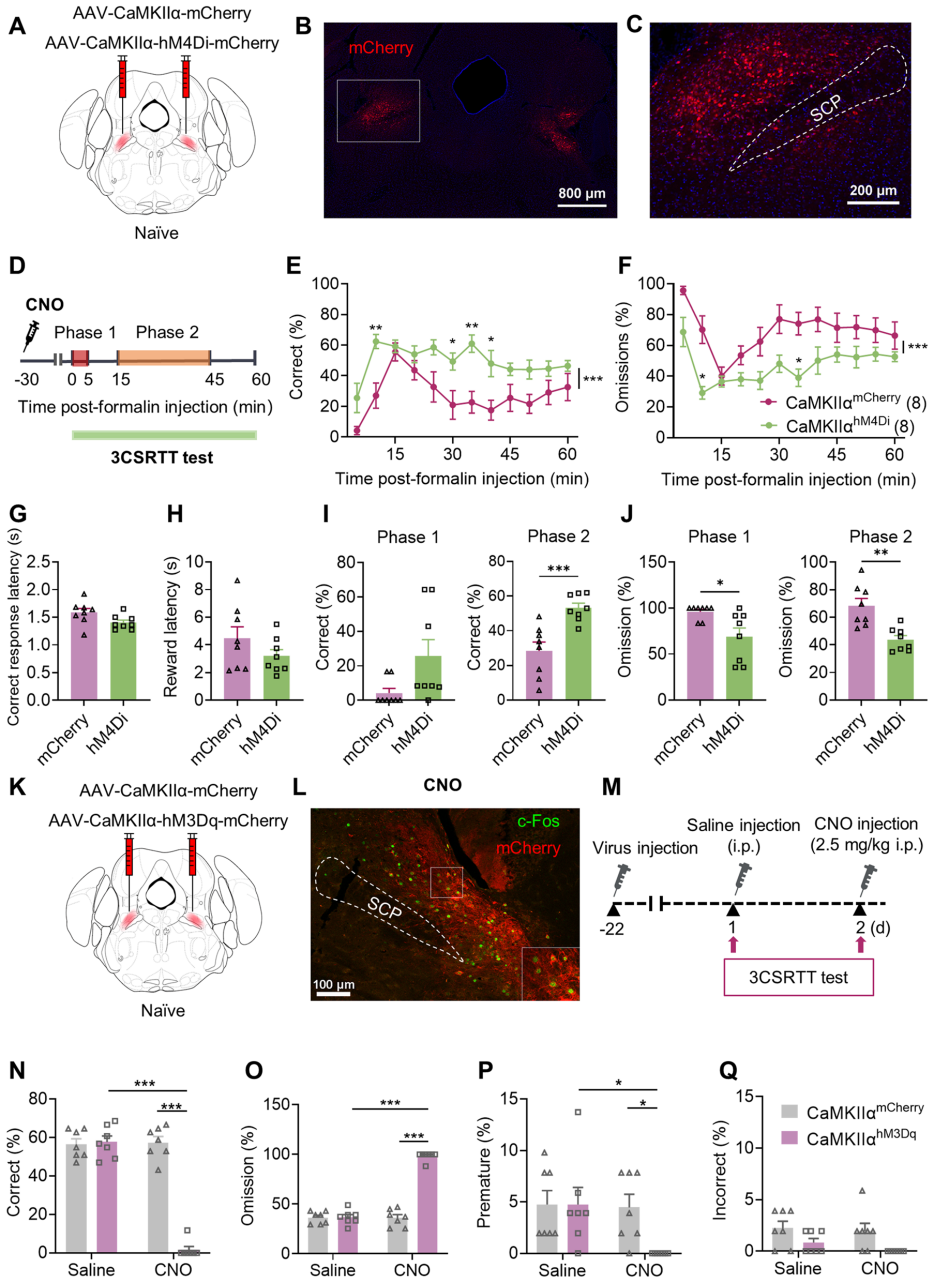
CaMKIIα neurons in the LPBN mediate the disruption of acute inflammatory pain on 3CSRTT performance. **A**–**J** The effect of chemogenetic inhibition of CaMKIIα neurons in the LPBN on acute inflammatory pain-induced 3CSRTT performance impairment. **A** Schematic of bilateral LPBN stereotaxic injection of AAV-CaMKIIα-mCherry or AAV-CaMKIIα-hM4Di-mCherry in naive mice. **B** Representative image of the restricted expression of AAV-CaMKIIα-hM4Di-mCherry in the bilateral LPBN. Scale bar, 800 μm. **C** Higher magnifications of the specified region in (**B**). Scale bar, 200 μm. **D** Experimental design. Mice were i.p. injected with CNO (2.5 mg/kg) 30 min before formalin intraplantar injection, and 3CSRTT was performed immediately after formalin injection for 60 min. **E**, **F** Correct (**E**) and omission (**F**) over the 60-min testing period in CaMKIIα^mCherry^ and CaMKIIα^hM4Di^ mice. Data points are displayed in 5-min time bins. **G**, **H** Correct response latency (**G**) and reward latency (**H**) in CaMKIIα^mCherry^ and CaMKIIα^hM4Di^ mice. **I**, **J** Correct (**I**) and omission (**J**) in phases 1 and 2 in CaMKIIα^mCherry^ and CaMKIIα^hM4Di^ mice (n = 7 mice per group). **K**–**Q** The effect of chemogenetic activation of CaMKIIα neurons in the LPBN on 3CSRTT performance. **K** Schematic of bilateral LPBN stereotaxic injection of AAV-CaMKIIα-mCherry or AAV-CaMKIIα-hM3Dq-mCherry in naive mice. **L** Representative image of the restricted expression of AAV-CaMKIIα-hM3Dq-mCherry in LPBN and CNO-evoked c-Fos expression in virally labelled neurons. **M** Experimental design. 3CSRTT (50 trials) was performed 30 min after saline i.p. injection on the first day. The next day, 3CSRTT was performed 30 min after CNO i.p. injection. **N**–**Q** Correct (**N**), omission (**O**), incorrect (**P**), and premature (**Q**) in the 3CSRTT in CaMKIIα^mCherry^ and CaMKIIα^hM3Dq^ mice (n = 7 mice per group). Data are mean ± SEM. *p < 0.05, **p < 0.01, ***p < 0.001. Two-way ANOVA followed by the Sidak’s multiple comparisons test in **E**, **F** and **N**–**Q**. Two-tailed unpaired t test in **G**–**J**

To illustrate the correlation between hyperexcitation of CaMKIIα neurons in the LPBN and the interruptive effect of pain on attention, we measured the effect of chemogenetic inhibition of CaMKIIα neurons in the LPBN on 3CSRTT performance following formalin injection. We induced repetitive pharmacogenetic inhibition of CaMKIIα neurons in the LPBN by intraperitoneal injection of CNO (2.5 mg/kg) 30 min before behavior tests in naive mice with the bilateral injection of AAV-CaMKIIα-hM4Di-mCherry (CaMKIIα^hM4Di^) or AAV-CaMKIIα-mCherry (CaMKIIα^mCherry^) into the LPBN. CaMKIIα^hM4Di^ mice showed significantly increased correct and decreased omission rates in Phase 2 (Fig. 5E, F, I and J). There were no significant differences in correct response latency, reward latency, incorrect response rate or premature response rate between CaMKIIα^mCherry^ mice and CaMKIIa^hM4Di^ mice (Fig. 5G, H; Additional file 1: Fig. S8). We demonstrated that chemogenetic inhibition of CaMKIIα neurons in the LPBN did not affect 3CSRTT performance in the physiological state (Additional file 1: Fig. S9). These findings suggest that inhibition of CaMKIIα neurons in the LPBN attenuates formalin-induced impairment of sustained-attention performance.

### Chemogenetic activation of CaMKIIα neurons in the LPBN mimics the acute inflammatory pain-induced impairment of 3CSRTT performance

To further illustrate the effect of activation of CaMKIIα neurons in the LPBN on 3CSRTT performance, we injected AAV-CaMKIIα-hM3Dq-mCherry virus bilaterally into the LPBN of naive mice to selectively activate LPBN ^CaMKIIα^ neurons (CaMKIIα^hM3Dq^) or AAV-CaMKIIα-mCherry as the control (CaMKIIα^mCherry^) (Fig. 5K, L). The 3CSRTT was performed 30 min after intraperitoneal injection of saline or CNO (Fig. 5M). We found that CaMKIIα^hM3Dq^ mice showed significantly increased omission rates and decreased correct rates. Six of seven of CaMKIIα^hM3Dq^ mice lost their ability to complete even a single trial of the 3CSRTT (Fig. 5N–Q). These findings suggest that activation of CaMKIIα neurons in the LPBN successfully mimics acute pain-induced impairment of 3CSRTT performance.

### CaMKIIα neurons in the LPBN mediate pain responses and pain-related aversion

**Fig. 6:**
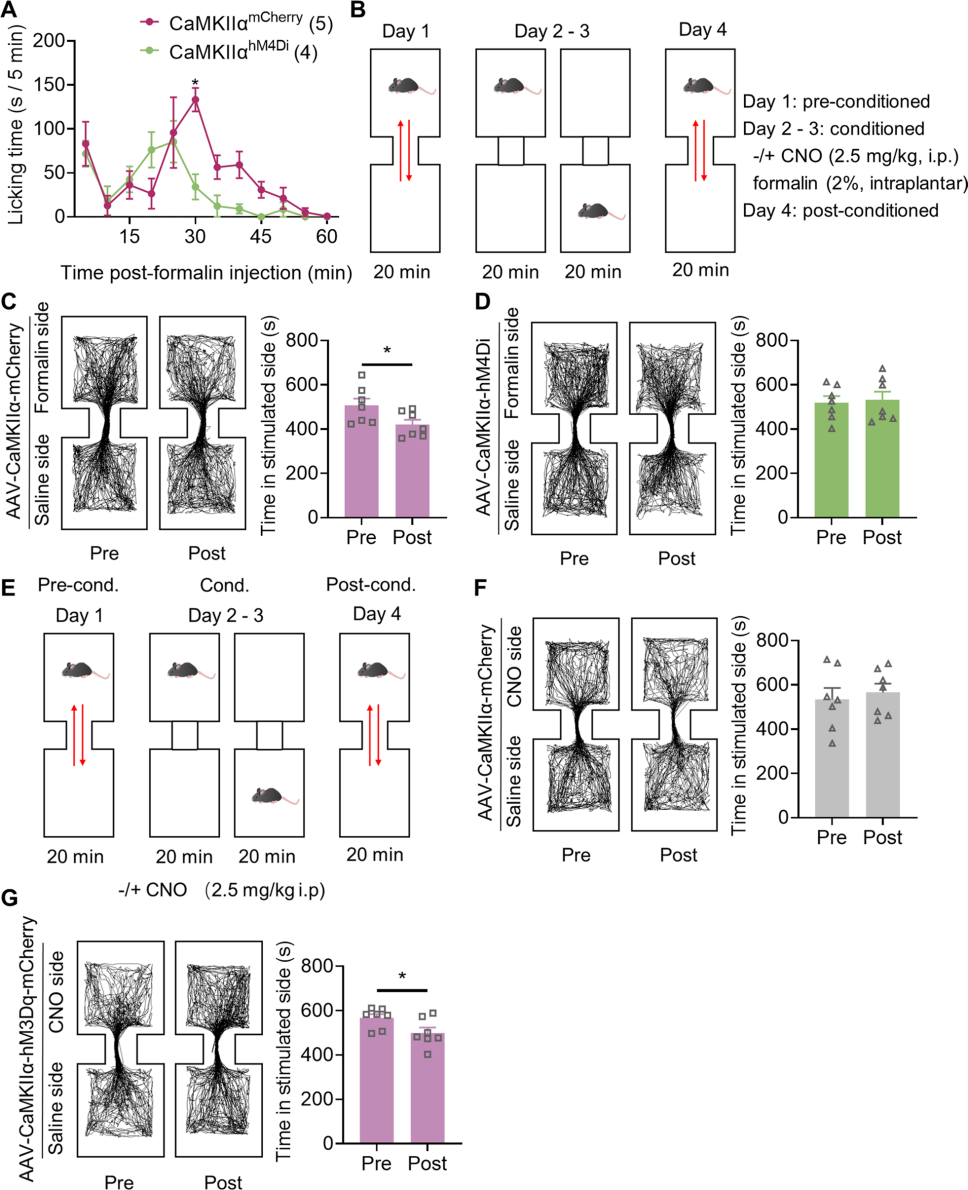
CaMKIIα neurons in the LPBN modulate pain responses and aversion. **A** Duration of licking in CaMKIIα^mCherry^ and CaMKIIα^hM4Di^ mice following formalin intraplantar injection. **B**–**D** The effect of chemogenetic inhibition of CaMKIIα neurons in the LPBN on CPA induced by intraplantar formalin injection. **B** Experimental design for CPA. CNO (i.p., 2.5 mg/kg) was given 30 min prior to 2% intraplantar formalin on Days 2 and 3, which was paired with one side of a two-chambered box differentiated by visual cues. **C**, **D** Examples of tracking maps and quantification of time spent in the stimulated side in the CPA before (Pre) (**C**) and after (Post) (**D**) injection of CNO into CaMKIIα^mCherry^ mice and CaMKIIα^hM4Di^ mice. **E**, **G** Chemogenetic activation of CaMKIIα neurons in the LPBN induced CPA. **E** Experimental design for CPA. CNO (i.p., 2.5 mg/kg) was given 30 min prior to training on Days 2 and 3, which was paired with one side of a two-chambered box differentiated by visual cues. **F**, **G** Examples of tracking maps and quantification of time spent in the stimulated side in the CPA before (Pre) (**F**) and after (Post) (**G**) injection of CNO into CaMKIIα^mCherry^ mice and CaMKIIα^hM3Dq^ mice. Data are mean ± SEM. *p < .05. Two-way ANOVA followed by the Sidak’s multiple comparisons test in **A**. Two-tailed unpaired t test in **C**, **D**, **F** and **G**

Formalin induced acute inflammatory pain leads to paw-licking behavior and pain-related aversion [24, 34]. To investigate whether CaMKIIα neurons in the LPBN modulated the 3CSRTT by influencing pain-related behaviors, we modulated CaMKIIα neurons in the LPBN by chemogenetics and assessed these behaviors. CaMKIIα^hM4Di^ mice showed significantly decreased paw-licking times during phase 2 following formalin injection (Fig. 6A). This alteration is consistent with the changes in 3CSRTT performance.

We then assessed whether CaMKIIα neurons in the LPBN modulated formalin-induced aversive memory using the conventional conditioned place aversion (CPA) behavioral paradigm. In the CPA test, CaMKIIα^mCherry^ mice showed CPA to a noxious stimulus while CaMKIIα^hM4Di^ mice attenuated learning this negative association (Fig. 6B–D). In the CNO-induced CPA test, CaMKIIα^hM3Dq^ mice showed CPA for the CNO-paired chamber while CaMKIIα^mCherry^ mice were unaffected by CNO administration (Fig. 6E–G). To investigate the effect of aversion on sustained-attention performance, an intraperitoneal injection of lithium chloride (LiCl, 0.15 mol/L, 2% body weight) was used to induce non-noxious aversion before 3CSRTT [35]. LiCl-injected mice showed a decreased rate of correct and an increased rate of omission in 3CSRTT and increased c-Fos expression in the LPBN (Additional file 1: Fig. S10). These results show that CaMKIIα neurons in the LPBN mediated pain-related aversion, and aversion itself can disrupt sustained-attention performance, suggesting that chemogenetic inhibition of CaMKIIα neurons in the LPBN may improve 3CSRTT performance under acute inflammatory pain by attenuating pain-related aversion.

### Insufficient activation of CaMKIIα neurons in the LPBN is correlated with the recovery of 3CSRTT performance during chronic inflammatory pain

**Fig. 7:**
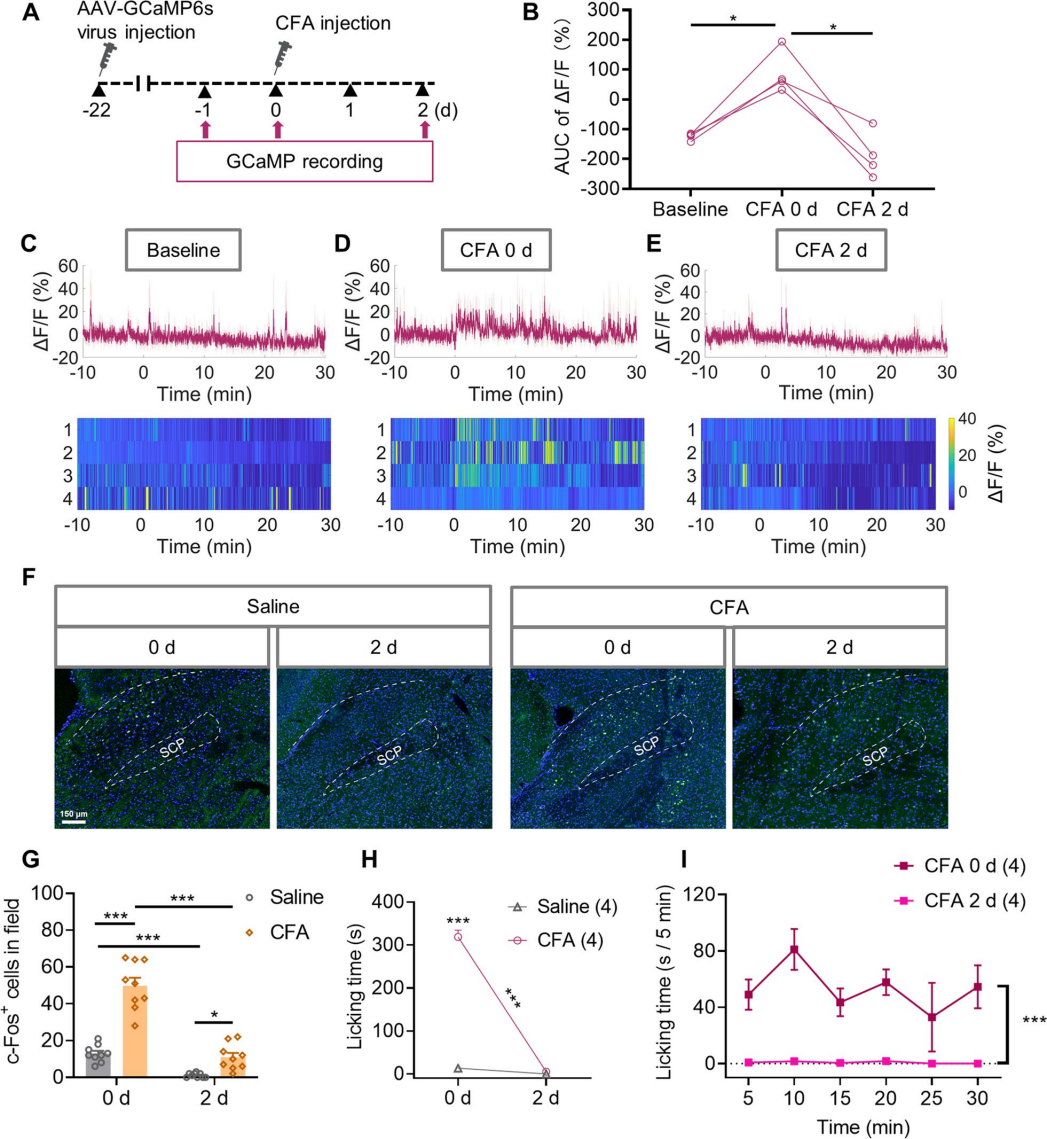
Insufficient activation of CaMKIIα neurons in the LPBN on Day 2 after CFA injection. **A**–**E** In vivo calcium imaging of CaMKIIα neurons in the LPBN at different time points. **A** Experimental design. AAV-CaMKIIα-GCaMP6s was injected into the LPBN 21 days before in vivo calcium imaging, and 40-min in vivo calcium imaging was performed 1 day before CFA injection (baseline), the day of CFA injection (CFA 0 d) and Day 2 after CFA injection (CFA 2 d) in awake mice. The recording was continued for 40 min at the same time each day and the recording began 10 min before the start of CFA injection on CFA 0 d. **B** Quantification of GCaMP6s signals at baseline, CFA 0 d and CFA 2 d in awake mice via AUC of ΔF/F during 0–30 min. **C**–**E** Average GCaMP6s signal and heatmap of ΔF/F (%) for all individual mice at baseline, CFA 0 d and CFA 2 d (n = 4 mice). **F**, **G** Graphs depicting quantification of c-Fos^+^ neurons in the LPBN and representative images following saline or CFA injection on the day of injection (0 d) and 2 d post injection (n = 9 sections from three mice per group). **H** Time spent licking the paw following saline or CFA injection on the day of injection (0 d) and 2 d post injection. The recording was continued for 30 min at the same time each day and was immediately performed following CFA injection on CFA 0 d. **I** Time spent licking paw following CFA injection CFA 0 d and CFA 2 d displayed in 5-min time bins. Data are mean ± SEM. *p < 0.05, **p < 0.01, ***p < 0.001. One-way ANOVA followed by the Tukey’s multiple comparisons test in **B**. Two-way ANOVA followed by the Sidak’s multiple comparisons test in **G-I**

The impairment of 3CSRTT performance was accompanied by increasing activity of CaMKIIα neurons in the LPBN in formalin-induced phases 1 and 2; however, both returned to normal during the interphase (Figs. 1D, E, 4F–H). Thus, we speculated that the different excitability of CaMKIIα neurons in the LPBN under acute or chronic inflammatory pain might correlate with the performance in the 3CSRTT. To test this hypothesis, we first investigate the difference in excitability of CaMKIIα neurons in the LPBN on the day of CFA injection (CFA 0 d) and on Day 2 after CFA injection (CFA 2 d) by measuring the calcium signals of CaMKIIα neurons in the LPBN (Fig. 7A). The Ca^2+^ responses at CFA 0 d were significantly higher than at the baseline (1 day before CFA injection), and returned to the baseline level at CFA 2 d, indicating significant increase activities of CaMKIIα neurons in the LPBN only on CFA 0 d (Fig. 7B–E). We then measured c-Fos expression in the LPBN following saline or CFA injection on the day of injection (0 d) and Day 2 after injection; c-Fos expression was more significant in CFA-injected mice on 0 d than in saline-injected mice, while c-Fos expression at CFA 2 d was significantly less than that at CFA 0 d but still higher than that at saline 2 d (Fig. 7F, G). The activation of CaMKIIα neurons in the LPBN is associated with formalin-induced paw-licking behavior (Fig. 6A). Therefore, we measured the duration of paw-licking behavior following saline or CFA injection on the day of injection and Day 2 after injection. The licking duration in the CFA-injected mice was much significant on 0 d than with saline-injected mice but almost disappeared on Day 2 after injection (Fig. 7H, I). These findings suggest that the excitability of CaMKIIα neurons in the LPBN at CFA 2 d was lower than at CFA 0 d.

**Fig. 8:**
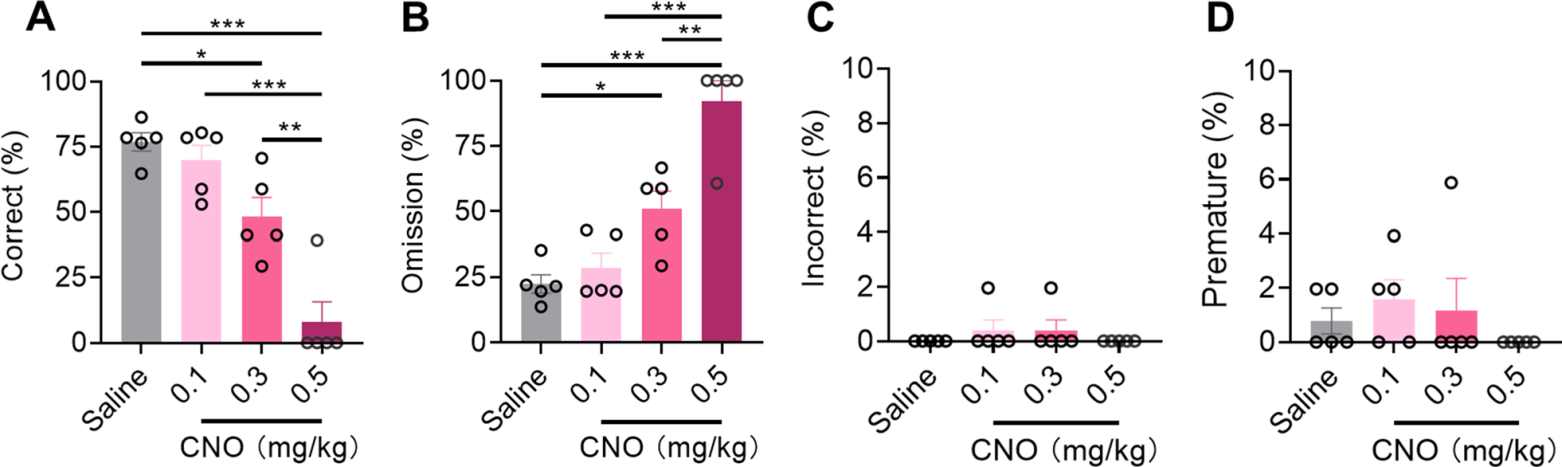
Activity of CaMKIIα neurons in the LPBN is correlated with the impairment of 3CSRTT performance. **A**–**D** Correct (**A**), omission (**B**), incorrect (**C**), and premature (**D**) in the 3CSRTT started 30 min after i.p. injection of either saline, 0.1, 0.3 or 0.5 mg/kg CNO (n = 5 mice per group). Data are mean ± SEM. *p < 0.05, **p < 0.01, ***p < 0.001. One-way ANOVA followed by the Tukey’s multiple comparisons test in **A**–**D**

To verify the correlation between the increased activity of CaMKIIα neurons in the LPBN and impairment of 3CSRTT performance, the 3CSRTT was initiated 30 min after i.p. injection of either 0.1, 0.3, or 0.5 mg/kg CNO in CaMKIIα^hM3Dq^ mice. With increasing CNO concentrations, the correct rate of 3CSRTT decreased significantly, accompanied by an increasing omission rate, while there were no significant differences in incorrect or premature rates (Fig. 8A–D). Together, these findings suggest that the reduced activation of CaMKIIα neurons in the LPBN is correlated with the recovery of 3CSRTT performance in the chronic phase of inflammatory pain.

## Discussion

**Fig. 9:**
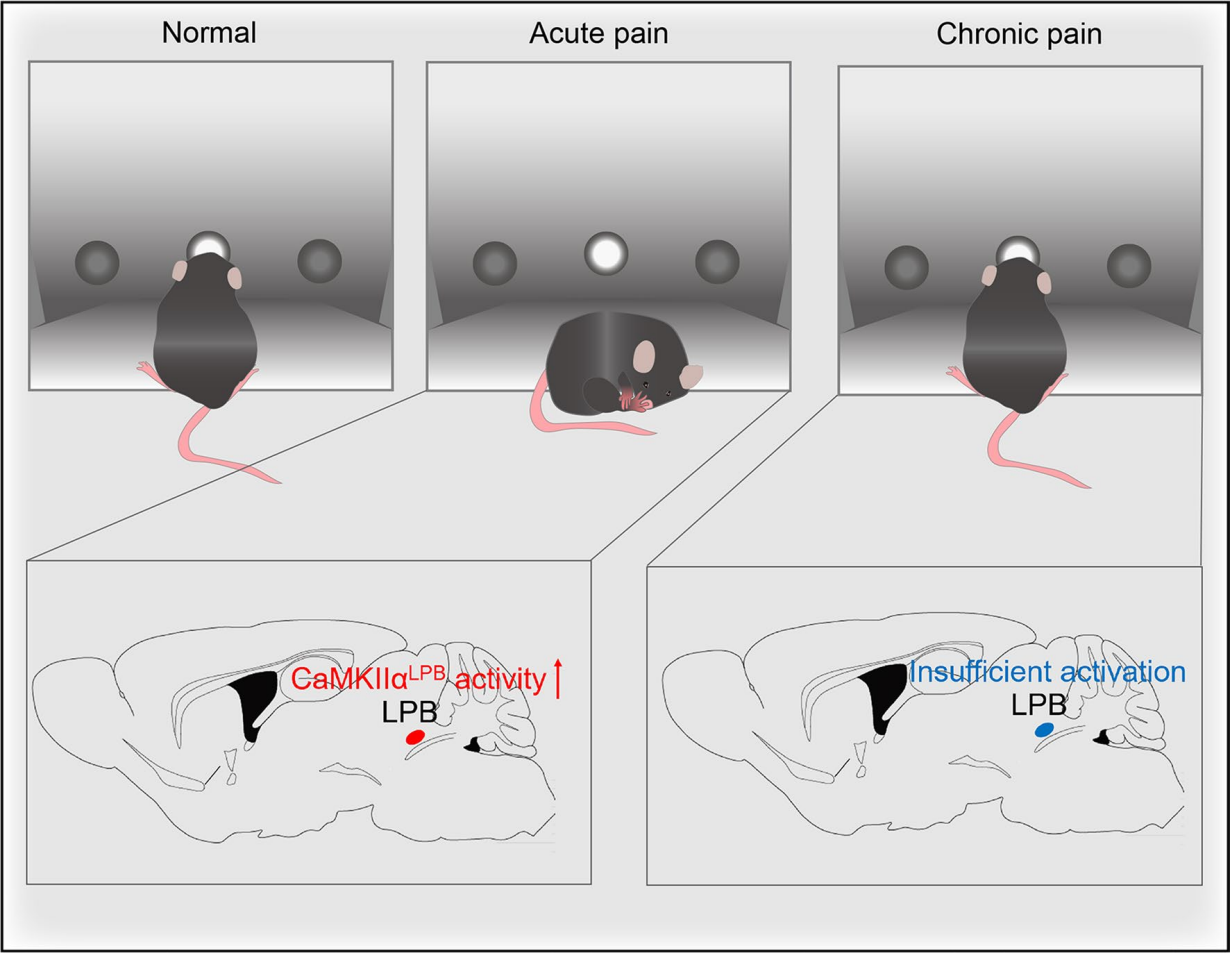
Schematic representation of the working model. Acute inflammatory pain impairs sustained attention performance as measured by the 3CSRTT, while chronic inflammatory pain has no significant effect. The hyperexcitation of CaMKIIα neurons in the LPBN contributes to the acute inflammatory pain-induced impairment of sustained attention performance in 3CSRTT. Insufficient activation of CaMKIIα neurons in the LPBN is correlated with the recovery of performance on the sustained-attention task during chronic inflammatory pain

We showed that acute inflammatory pain impairs sustained-attention performance measured by the 3CSRTT, while chronic inflammatory pain has no significant effect. The hyperexcitation of CaMKIIα neurons in the LPBN contributes to the acute inflammatory pain-induced impairment of sustained attention performance in 3CSRTT. Insufficient activation of CaMKIIα neurons in the LPBN under chronic inflammatory pain mediates the recovery of performance on the sustained-attention task (Fig. 9).

Clinical studies show that sustained attention is vulnerable to acute pain [16]. Sustained-attention impairment has also been observed in patients with chronic pain [36]. However, recent studies have demonstrated that chronic pain is more likely to preserve performance on sustained tasks while impairing dual-task performance by orienting subjects to one task or the other without being able to maintain both [3]. These findings suggest that the effect of acute pain on attention differs from that of chronic pain. Our results confirm that acute nociceptive input leads to impaired performance on a sustained-attention task, but a chronic inflammatory pain state does not, providing behavioral evidence for the conclusions of the clinical studies.

Human studies suggest an overlap between the neuro-anatomical cerebral circuits of pain and attention. Our results show that acute inflammatory pain impairs 3CSRTT performance in phases 1 and 2 but does not affect performance in the interphase. The interphase of the formalin test is caused by the hyperpolarization of peripheral nociceptor TRPA1, suggesting that nociceptive input is paused during this period [37]. Therefore, our results suggest that the changes in 3CSRTT behavior are highly dependent on ascending pain pathways. Furthermore, our findings show that blocking the ascending sensory signal of the sciatic nerve can attenuate the effect of acute inflammatory pain on 3CSRTT, confirming the critical role of ascending pain pathways in the interruptive effect of pain on attention. However, the limited retention time of lidocaine is insufficiently long to cover phases 1 and 2, which is a limitation of this experiment. Our findings suggest that the neuro-anatomical cerebral circuits of pain and attention overlap in ascending pain pathways.

The LPBN is a crucial component of the ascending pain pathway. The LPBN sends projections to several brain regions, and some of these regions are directly or indirectly involved in attention regulation, such as the amygdala, the ventral tegmental area and the mediodorsal thalamus [21, 24, 28, 38–40]. Therefore, pain-induced hyperexcitation of excitatory neurons in the LPBN may directly impair performance on attentional tasks. Our findings suggest that the increased activity of CaMKIIα neurons in the LPBN impairs 3CSRTT performance through an increase in omissions, and inhibition of this neuronal cluster attenuates the impairment of 3CSRTT performance, demonstrating that the increased activity of CaMKIIα neurons in the LPBN induced by acute inflammatory pain is sufficient for disrupting the performance of attentional tasks. We also found that inhibition of CaMKIIα neurons in the LPBN significantly attenuated the impairment of 3CSRTT performance in phase 2 but not phase 1, suggesting that other neural circuits mediate the conduction of pain in phase 1, and providing evidence that acute pain may consume more attentional resources.

Previous studies demonstrated that the activity of excitatory neurons in the LPBN correlates with the intensity of noxious stimuli [41]. Our results demonstrate that the excitability of CaMKIIα neurons in the LPBN was lower in interphase and 45–60 min than in phases 1 and 2 following formalin injection, consistent with the changes in 3CSRTT performance in those periods. Therefore, we suppose that the intensity of spontaneous noxious stimuli input from the injured area is less under chronic inflammatory pain than with acute inflammatory pain; therefore, the LPBN will not be sufficiently activated to attract excessive attentional resources. A previous study has shown that pinch-induced Ca^2+^ responses in LPBN excitatory neurons are elevated in chronic pain states compared to the normal state [20]. However, pinch-induced acute nociceptive input cannot reflect inflammation-evoked spontaneous nociceptive input. Our findings suggest that the activation of CaMKIIα neurons in the LPBN was significantly lower on Day 2 after CFA injection than immediately after CFA injection, and the insufficient activation of CaMKIIα neurons in the LPBN correlated with the recovery of 3CSRTT performance. These findings support our hypotheses, suggesting that the spontaneous excitability of excitatory neurons in the LPBN is lower under chronic inflammatory pain than under acute inflammatory pain. Further studies are needed to elucidate the mechanisms of the difference in the excitability of excitatory neurons in the LPBN between acute and chronic pain states in the CFA model.

Clinical studies have shown that non-painful aversive stimuli, such as aversive white noise or money loss, can also produce similar attentional bias effects as painful stimuli [42]. In agreement, we have demonstrated the interruptive effect of non-painful aversive stimuli on attentional performance by LiCl injection. This finding suggests that acute inflammatory pain-induced aversion may participate in the disruption of sustained-attention performance. It should be noted that our findings demonstrate that the effects of chemogenetic regulation of CaMKIIα neurons in the LPBN on 3CSRTT performance are correlated with changes in pain-related aversion. Therefore, the effects of CaMKIIα neurons in the LPBN on attention may be exerted through downstream brain regions involved in the regulation of aversion.

## Conclusions

In summary, our findings indicate that the mechanisms by which acute inflammatory pain disrupts sustained attention are in part mediated by activation of ascending pain pathways and excitatory neurons in the LPBN, and insufficient activation of CaMKIIα neurons in the LPBN is correlated with the absence of significant impairment in 3CSRTT performance during chronic inflammatory pain. This finding has implications for the treatment of pain and its cognitive comorbidities.

### Supplementary Information

**Additional file 1:** **Figure S1.** Training for the 3CSRTT. **Figure S2.** Formalin-induced acute inflammatory pain has no effect on 3CSRTT performance on Day 1 after injection. **Figure S3.** Intraplantar injection of saline has no significant effect on 3CSRTT performance. **Figure S4.** Intraplantar injection of saline has no significant effect on 3CSRTT performance. **Figure S5.** Formalin paw injection has no significant effect on food intake over 1 h after the food pallet is given. **Figure S6.** Lidocaine injection into the popliteal space has no significant effect on 3CSRTT performance. **Figure S7.** Lidocaine-induced sciatic nerve blockade increases incorrect and premature responses in the 3CSRTT following formalin injection. **Figure S8.** Chemogenetic inhibition of LPBN CaMKIIα neurons has no effect on incorrect or premature responses in the 3CSRTT following formalin injection. **Figure S9.** Chemogenetic inhibition of LPBN CaMKIIα neurons has no effect on 3CSRTT performance in the normal state. **Figure S10.** Intraperitoneal injection of lithium chloride (LiCl) impairs 3CSRTT performance.

**Additional file 2:** **Table S1.** Details of the statistical analyses.

## Data Availability

All data generated or analyzed during this study are included in this published article and its supplementary information files. The datasets used and/or analyzed during the current study are available from the corresponding author upon reasonable request.

## Abbreviations

3CSRTT: Three-choice serial reaction time task
AAV: Adeno-associated virus
ANOVA: Analysis of variance
CaMKIIα: Ca^2+^/calmodulin-dependent protein kinase IIα
CFA: Complete Freund's adjuvant
CNO: Clozapine-N-oxide
CPA: Conditioned place aversion
EGFP: Enhanced green fluorescent protein
LiCl: Lithium chloride
LPBN: Lateral parabrachial nucleus
SCP: Superior cerebellar peduncle

## References

[1] Dumoulin S, Bouchard S, Loranger C, Quintana P, Gougeon V, Lavoie KL (2020). Are cognitive load and focus of attention differentially involved in pain management: an experimental study using a cold pressor test and virtual reality. J Pain Res.

[2] Eccleston C, Crombez G (1999). Pain demands attention: a cognitive-affective model of the interruptive function of pain. Psychol Bull.

[3] Moore DJ, Meints SM, Lazaridou A, Johnson D, Franceschelli O, Cornelius M (2019). The effect of induced and chronic pain on attention. J Pain.

[4] Landro NI, Fors EA, Vapenstad LL, Holthe O, Stiles TC, Borchgrevink PC (2013). The extent of neurocognitive dysfunction in a multidisciplinary pain centre population. Is there a relation between reported and tested neuropsychological functioning?. Pain.

[5] Moriarty O, McGuire BE, Finn DP (2011). The effect of pain on cognitive function: a review of clinical and preclinical research. Prog Neurobiol.

[6] Gong W, Fan L, Luo F (2019). Does experimentally induced pain affect attention? A meta-analytical review. J Pain Res.

[7] Mifflin K, Chorney J, Dick B (2016). Attention and working memory in female adolescents with chronic pain and pain-free female adolescents: a preliminary pilot study. Clin J Pain.

[8] Corti EJ, Gasson N, Loftus AM (2021). Cognitive profile and mild cognitive impairment in people with chronic lower back pain. Brain Cogn.

[9] Dick B, Eccleston C, Crombez G (2002). Attentional functioning in fibromyalgia, rheumatoid arthritis, and musculoskeletal pain patients. Arthritis Rheum.

[10] Khera T, Rangasamy V (2021). Cognition and pain: a review. Front Psychol.

[11] Boyette-Davis JA, Thompson CD, Fuchs PN (2008). Alterations in attentional mechanisms in response to acute inflammatory pain and morphine administration. Neuroscience.

[12] Martin TJ, Strassburg TJ, Grigg AL, Kim SA, Ririe DG, Eisenach JC (2017). Assessment of behavioral disruption in rats with abdominal inflammation using visual cue titration and the five-choice serial-reaction time task. Anesthesiology.

[13] Ririe DG, Boada MD, MacGregor MK, Martin SJ, Strassburg TJ, Kim SA (2018). Incisional nociceptive input impairs attention-related behavior and is associated with reduced neuronal activity in the prefrontal cortex in rats. Anesthesiology.

[14] Higgins GA, Silenieks LB, Van Niekerk A, Desnoyer J, Patrick A, Lau W (2015). Enduring attentional deficits in rats treated with a peripheral nerve injury. Behav Brain Res.

[15] Seminowicz DA, Davis KD (2007). Pain enhances functional connectivity of a brain network evoked by performance of a cognitive task. J Neurophysiol.

[16] Buffington AL, Hanlon CA, McKeown MJ (2005). Acute and persistent pain modulation of attention-related anterior cingulate fMRI activations. Pain.

[17] Yoshino A, Otsuru N, Okada G, Tanaka K, Yokoyama S, Okamoto Y (2021). Brain changes associated with impaired attention function in chronic pain. Brain Cogn.

[18] Huang J, Gadotti VM, Chen L, Souza IA, Huang S, Wang D (2019). A neuronal circuit for activating descending modulation of neuropathic pain. Nat Neurosci.

[19] Wang LH, Ding WQ, Sun YG (2022). Spinal ascending pathways for somatosensory information processing. Trends Neurosci.

[20] Sun L, Liu R, Guo F, Wen MQ, Ma XL, Li KY (2020). Parabrachial nucleus circuit governs neuropathic pain-like behavior. Nat Commun.

[21] Yang H, de Jong JW, Cerniauskas I, Peck JR, Lim BK, Gong H (2021). Pain modulates dopamine neurons via a spinal-parabrachial-mesencephalic circuit. Nat Neurosci.

[22] Zhang L, Wang J, Niu C, Zhang Y, Zhu T, Huang D (2021). Activation of parabrachial nucleus—ventral tegmental area pathway underlies the comorbid depression in chronic neuropathic pain in mice. Cell Rep.

[23] Palmiter RD (2018). The parabrachial nucleus: CGRP neurons function as a general alarm. Trends Neurosci.

[24] Chiang MC, Nguyen EK, Canto-Bustos M, Papale AE, Oswald AM, Ross SE (2020). Divergent neural pathways emanating from the lateral parabrachial nucleus mediate distinct components of the pain response. Neuron.

[25] Chiang MC, Bowen A, Schier LA, Tupone D, Uddin O, Heinricher MM (2019). Parabrachial complex: a hub for pain and aversion. J Neurosci.

[26] Holland PC, Han JS, Gallagher M (2000). Lesions of the amygdala central nucleus alter performance on a selective attention task. J Neurosci.

[27] Rabellino D, Densmore M, Harricharan S, Jean T, McKinnon MC, Lanius RA (2018). Resting-state functional connectivity of the bed nucleus of the stria terminalis in post-traumatic stress disorder and its dissociative subtype. Hum Brain Mapp.

[28] Edelstyn NM, Mayes AR, Ellis SJ (2014). Damage to the dorsomedial thalamic nucleus, central lateral intralaminar thalamic nucleus, and midline thalamic nuclei on the right-side impair executive function and attention under conditions of high demand but not low demand. Neurocase.

[29] Kim H, Ahrlund-Richter S, Wang X, Deisseroth K, Carlen M (2016). Prefrontal parvalbumin neurons in control of attention. Cell.

[30] Leszczynska K, Kau ST (1992). A sciatic nerve blockade method to differentiate drug-induced local anesthesia from neuromuscular blockade in mice. J Pharmacol Toxicol Methods.

[31] Lim TK, Macleod BA, Ries CR, Schwarz SK (2007). The quaternary lidocaine derivative, QX-314, produces long-lasting local anesthesia in animal models in vivo. Anesthesiology.

[32] Robbins TW (2002). The 5-choice serial reaction time task: behavioural pharmacology and functional neurochemistry. Psychopharmacology.

[33] Gong W, Li J, Luo F (2020). Time course of attention interruption after transient pain stimulation. J Pain.

[34] Rosland JH, Tjolsen A, Maehle B, Hole K (1990). The formalin test in mice: effect of formalin concentration. Pain.

[35] Bagdas D, Muldoon PP, AlSharari S, Carroll FI, Negus SS, Damaj MI (2016). Expression and pharmacological modulation of visceral pain-induced conditioned place aversion in mice. Neuropharmacology.

[36] van der Leeuw G, Leveille SG, Dong Z, Shi L, Habtemariam D, Milberg W (2018). Chronic pain and attention in older community-dwelling adults. J Am Geriatr Soc.

[37] Fischer M, Carli G, Raboisson P, Reeh P (2014). The interphase of the formalin test. Pain.

[38] Dal Monte O, Costa VD, Noble PL, Murray EA, Averbeck BB (2015). Amygdala lesions in rhesus macaques decrease attention to threat. Nat Commun.

[39] Flores-Dourojeanni JP, van Rijt C, van den Munkhof MH, Boekhoudt L, Luijendijk MCM, Vanderschuren L (2021). Temporally specific roles of ventral tegmental area projections to the nucleus accumbens and prefrontal cortex in attention and impulse control. J Neurosci.

[40] Huang T, Lin SH, Malewicz NM, Zhang Y, Zhang Y, Goulding M (2019). Identifying the pathways required for coping behaviours associated with sustained pain. Nature.

[41] Campos CA, Bowen AJ, Roman CW, Palmiter RD (2018). Encoding of danger by parabrachial CGRP neurons. Nature..

[42] Britton MK, Anderson BA (2021). Attentional avoidance of threatening stimuli. Psychol Res.

