# Correction: Excitatory neurons in the lateral parabrachial nucleus mediate the interruptive effect of inflammatory pain on a sustained attention task

**DOI:** 10.1186/s12967-024-04935-z

**Published:** 2024-02-29

**Authors:** Huan-Yu Zheng, Yu-Meng Chen, Yao Xu, Cheng Cen, Yun Wang

**Affiliations:** 1 Neuroscience Research Institute and Department of Neurobiology, School of Basic Medical Sciences, Key Laboratory for Neuroscience, Ministry of Education/National Health Commission and State Key Laboratory of Natural and Biomimetic Drugs, Peking University, Beijing, 100191 China; 2 PKU‑IDG/McGovern Institute for Brain Research, Peking University, Beijing, 100871 China

**Correction: Journal of Translational Medicine (2023) 21:896** 10.1186/s12967-023-04583-9

Following publication of the original article [1], we have been notified that Dr. Cheng Cen was not mentioned as one of the corresponding authors. She should also be the corresponding author as per below:

Cheng Cen1*, ORCID 0000-0001-5364-0245

The original article was updated.
