# Supplementary material for: Excitatory neurons in the lateral parabrachial nucleus mediate the interruptive effect of inflammatory pain on a sustained attention task
Source: J Transl Med. 2023 Dec 10;21:896. doi: 10.1186/s12967-023-04583-9 (PMC10712130; doi:10.1186/s12967-023-04583-9)
Supplement: Supplementary file 1 — Additional file 1: Figure S1. Training for the 3CSRTT. Figure S2. Formalin-induced acute inflammatory pain has no effect on 3CSRTT performance on Day 1 after injection. Figure S3. Intraplantar injection of saline has no significant effect on 3CSRTT performance. Figure S4. Intraplantar injection of saline has no significant effect on 3CSRTT performance. Figure S5. Formalin paw injection has no significant effect on food intake over 1 h after the food pallet is given. Figure S6. Lidocaine injection into the popliteal space has no significant effect on 3CSRTT performance. Figure S7. Lidocaine-induced sciatic nerve blockade increases incorrect and premature responses in the 3CSRTT following formalin injection. Figure S8. Chemogenetic inhibition of LPBN CaMKIIα neurons has no effect on incorrect or premature responses in the 3CSRTT following formalin injection. Figure S9. Chemogenetic inhibition of LPBN CaMKIIα neurons has no effect on 3CSRTT performance in the normal state. Figure S10. Intraperitoneal injection of lithium chloride (LiCl) impairs 3CSRTT performance. [file 12967_2023_4583_MOESM1_ESM.docx]

**
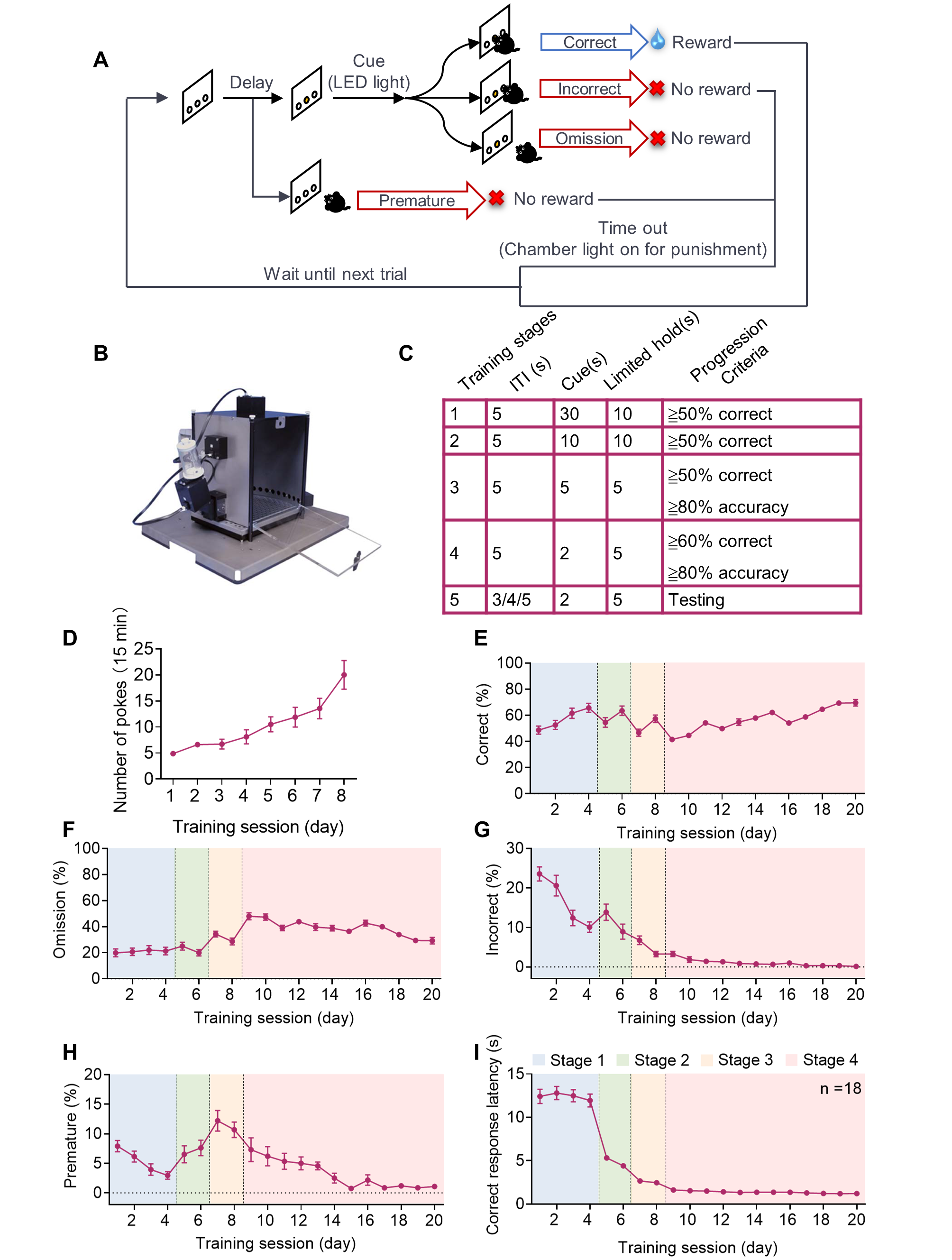
**

**Fig. S1:** **Training for the 3CSRTT. A** Schematic of the 3CSRTT for fully trained animals. **B** Photograph of the 3CSRTT apparatus. **C** Training in the 3CSRTT. The animals were trained at four levels defined by specific criteria. The criteria of each level had to be met for two consecutive days for progression to the next level. After successfully reaching the target criteria. After successfully reaching the target criteria (= level 4, two consecutive days with ≥ 60%, ≥ 80% accuracy, 2 s cue, 5 s maximum response time) the animals were used to study the effect of pain on attention. **D** Pretraining data for the animals used. **E-I** Training data for the animals used (n =18). 3CSRTT, three-choice serial reaction time task.

**
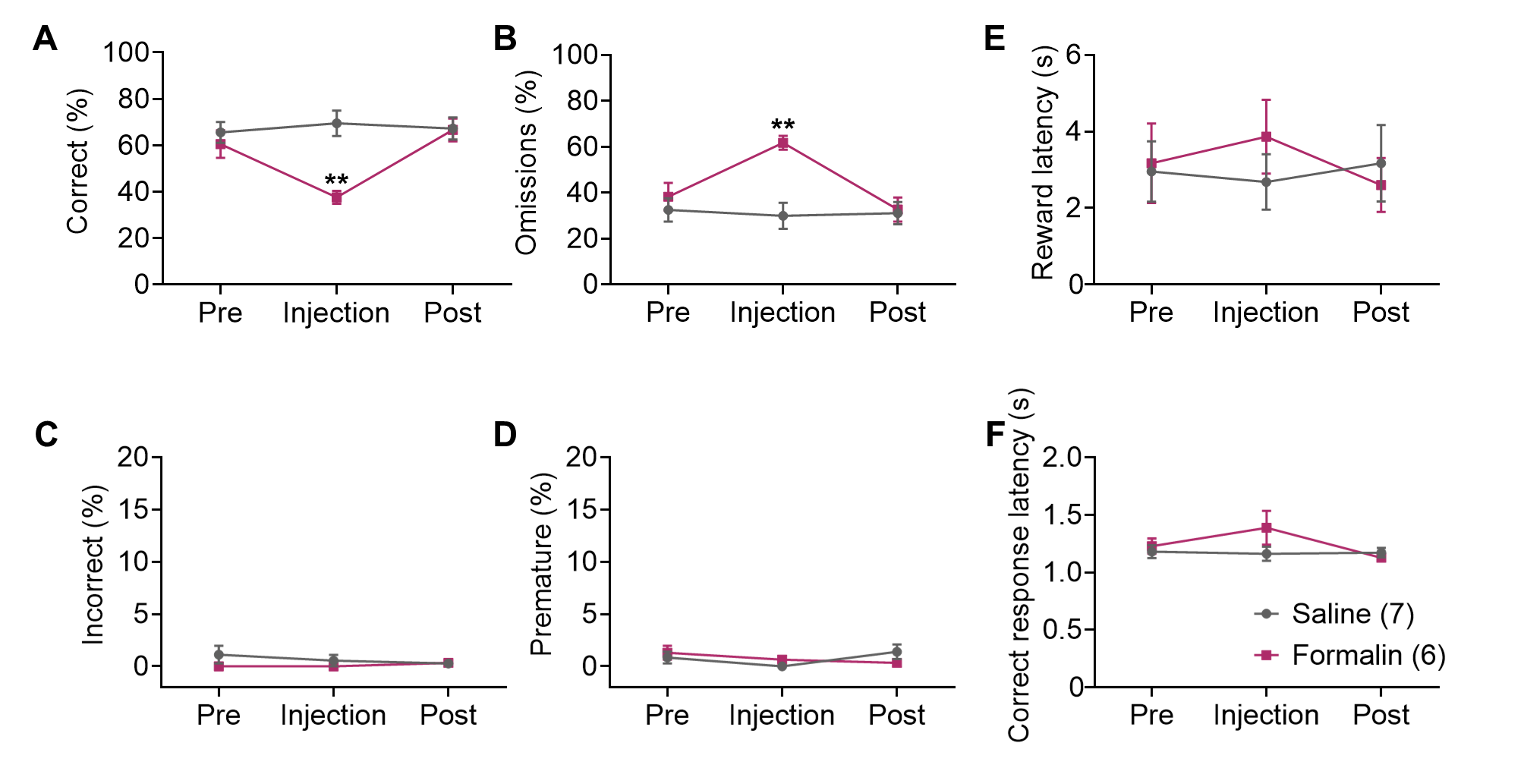
**

**Fig. S2: Formalin-induced acute inflammatory pain has no effect on 3CSRTT performance on Day 1 after injection. A-F** Effects of saline (n=7 mice) or formalin injection (n=6 mice) on correct rate, omission rate, incorrect rate, premature rate, reward latency and correct response latency in the 3CSRTT 1 day before injection (Pre), on the day of injection (Injection) and Day 1 after injection (Post). Data are mean ± SEM. ***p* < 0.01. Two-way analysis of variance followed by the Sidak’s multiple comparisons test in **A-F**. 3CSRTT, three-choice serial reaction time task.

**
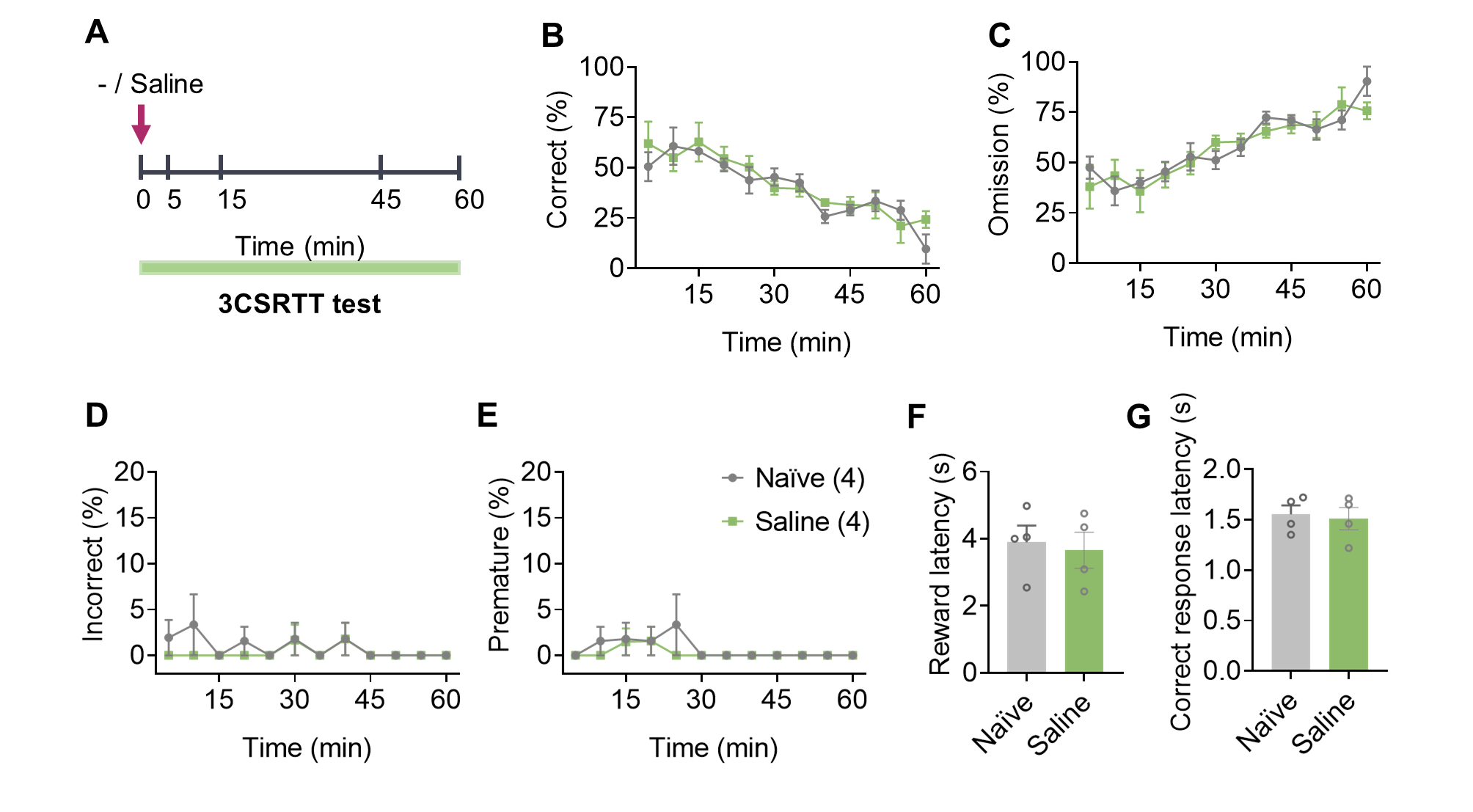
**

**Fig. S3:** **Intraplantar injection of saline has no significant effect on 3CSRTT performance. A** Experimental design. 3CSRTT was performed immediately without any treatment or after saline injection for 60 min. **B-E** Effects of saline injection on correct, omission, incorrect and premature responses in the 3CSRTT during the 60-min testing period compared with naïve group. Data points were displayed in 5-min time bins. **F, G** Effects of saline injection on reward latency and correct response latency in the 3CSRTT during the 60-min testing period compared with naïve group (n = 4 per group). Data are mean ± SEM. Two-way analysis of variance followed by the Sidak’s multiple comparisons test in **B-E**. Two-tailed unpaired t test in **F and G**. 3CSRTT, three-choice serial reaction time task.

**
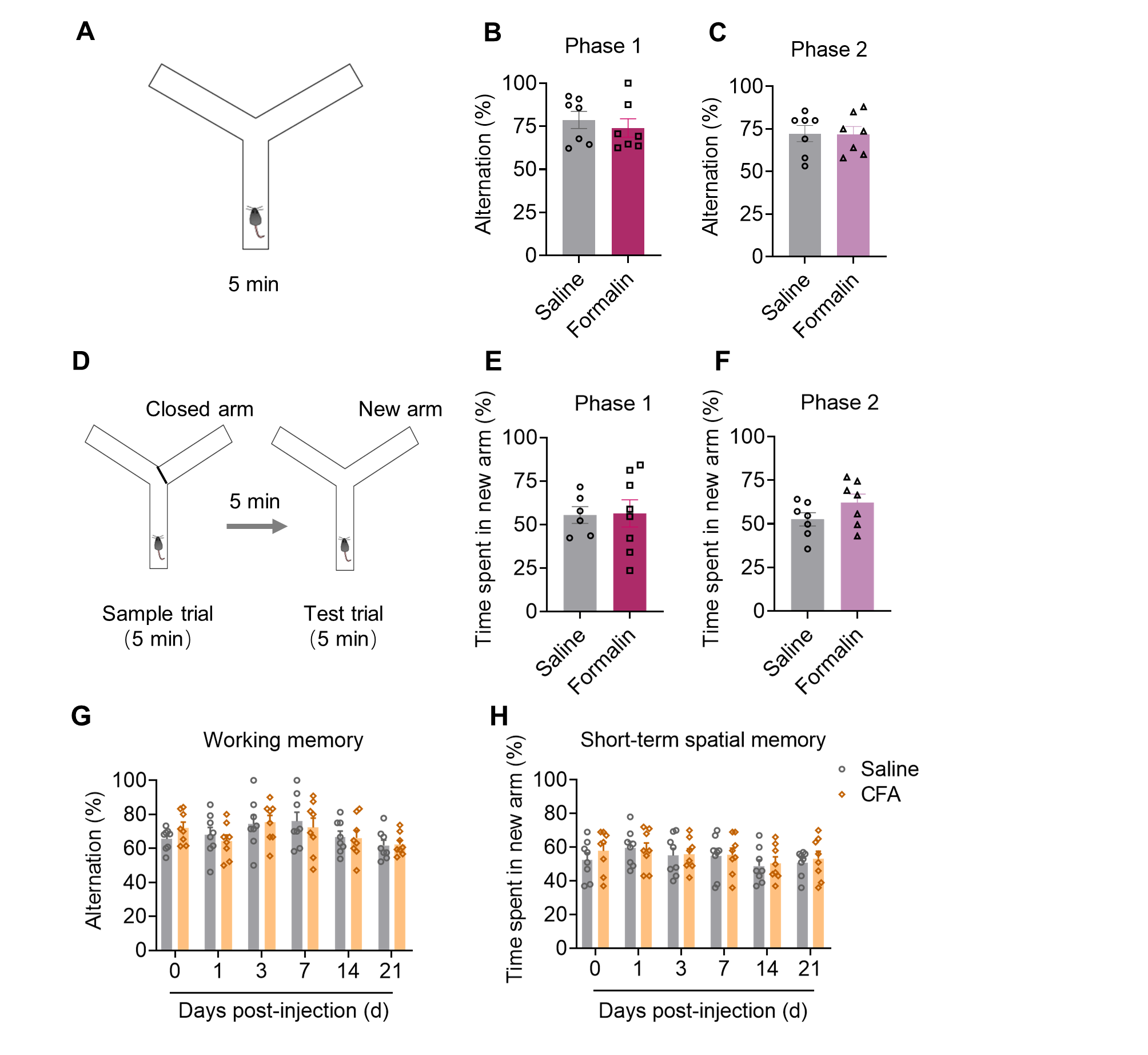
**

**Fig. S4:** **Intraplantar injection of saline has no significant effect on 3CSRTT performance. A** Schematic of Spontaneous Alternation test for spatial working memory. The test mouse was placed at the end of one arm and was allowed to explore freely for 5 minutes while spontaneous alternation behaviors were recorded and analyzed. **B, C** Effects of saline or formalin injection on alternation in phase 1 (n = 7 per group) and phase 2 (n = 7 per group). **D** Schematic of Blocked Arm test for short-term spatial memory. One of the three arms was blocked, the test mouse was allowed to explore the 2 unblocked arms for 5 min followed by rest for 5 min. The test mouse was returned to the maze with all 3 arms open and allowed to explore for another 5 min and time spent in the new arm was recorded. **E, F** Effects of saline or formalin injection on time spent in the new arm in phase 1 (n = 6 for saline group; n = 8 for formalin group) and phase 2 (n = 7 per group). **G** Effects of saline or CFA injection on alternation (n = 8 per group) and **(H)** time spent in the new arm (n = 8 per group) on the day immediately after injection as well as at 1 d, 3 d, 7 d, 14 d and 21 d post injection. Data are mean ± SEM. Two-tailed unpaired t test in **C-F**. Two-way analysis of variance followed by the Sidak’s multiple comparisons test in **G and H**. CFA, complete Freund's adjuvant.


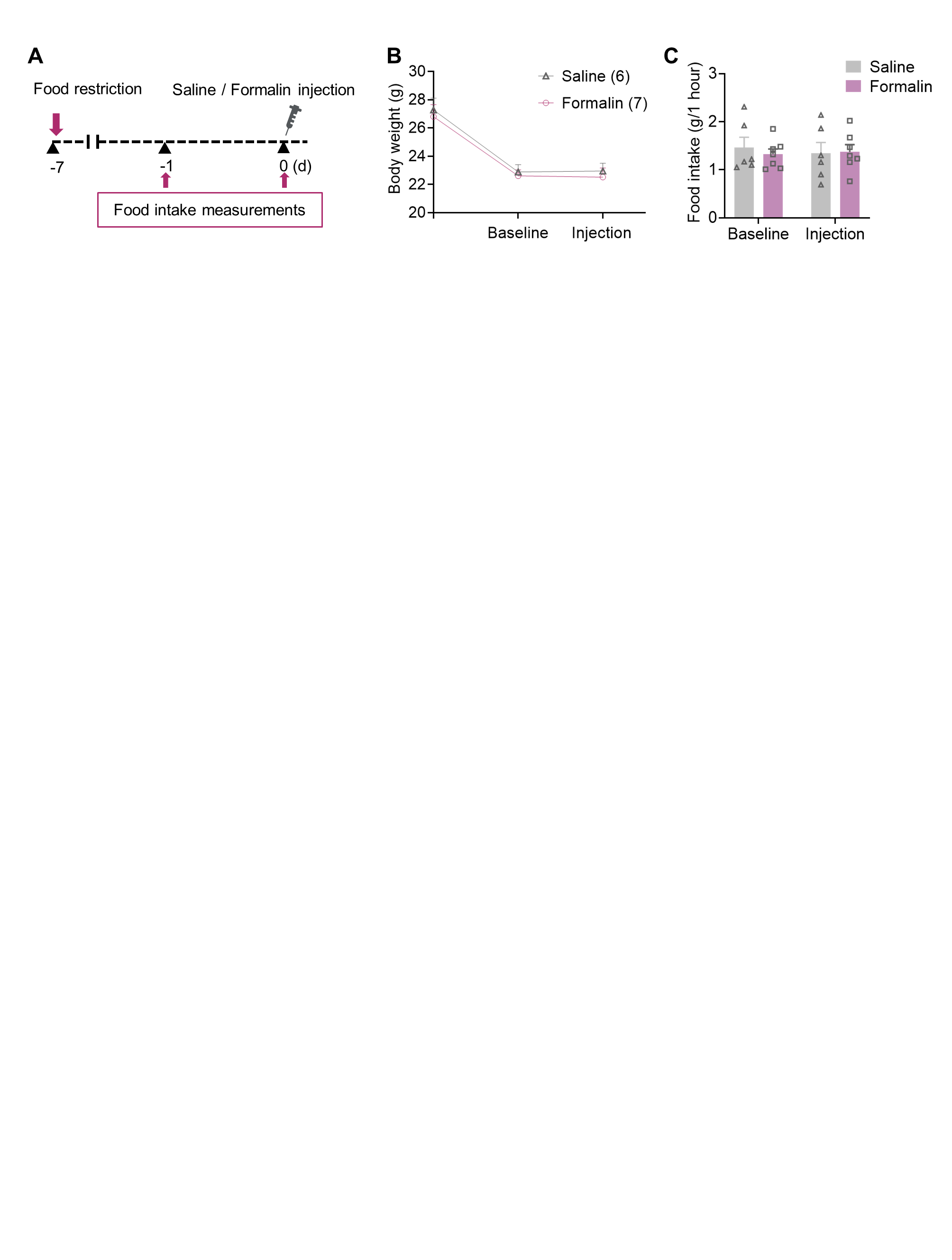


**Fig. S5: Formalin paw injection has no significant effect on food intake** **over 1 hour after the food pallet is given. A** Experimental design. Mice were put on a food restriction diet for a week to reduce their weight to 85%–90% of their starting body weight. Then, food intake was measured for 1 h after the food pallet was given to establish a baseline. The next day, mice were subcutaneously injected with saline or formalin into a hind paw, and food intake was measured in a manner consistent with the previous day. **B** The starting body weight and the body weight before food intake measurements of saline-injected group (n=6 mice) and formalin-injected group (n=7 mice). **C** Food intake on the baseline (-1 d) and the day of injection (0 d) of saline-injected mice and formalin-injected mice. Data are mean ± SEM. Two-way analysis of variance followed by the Sidak’s multiple comparisons test in **B-C**.

**
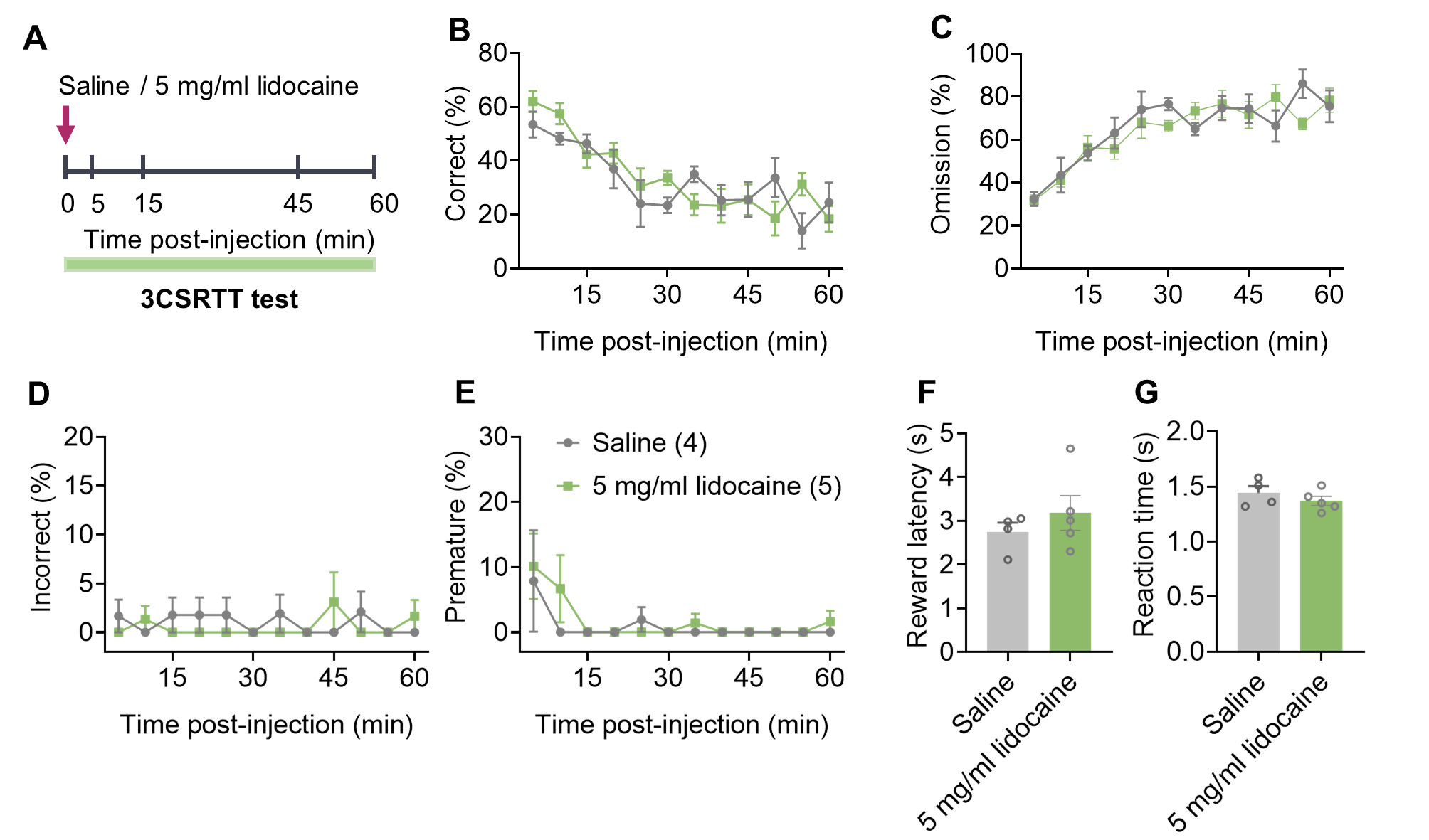
**

**Fig. S6:** **Lidocaine injection into popliteal space has no significant effect on 3CSRTT performance. A** Experimental design. 3CSRTT was performed immediately after either saline or lidocaine into the popliteal space for 60 min. **B-E** Effects of saline or lidocaine injection in the popliteal space on correct, omission, incorrect and premature responses in the 3CSRTT during the 60-min testing period. Data points are displayed in 5-min time bins. **F, G** Effects of saline or lidocaine injection on reward latency and correct response latency in the 3CSRTT during the 60-min testing period. Data are mean ± SEM. Two-way analysis of variance followed by the Sidak’s multiple comparisons test in **B-E**. Two-tailed unpaired t test in **F and G**. 3CSRTT, three-choice serial reaction time task.

**
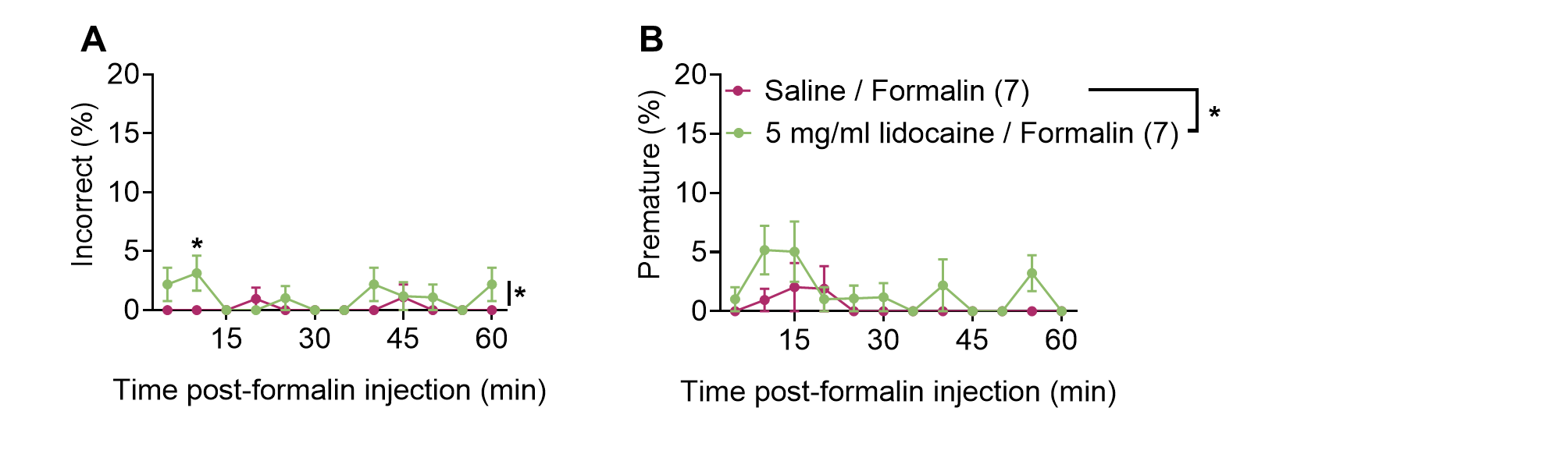
**

**Fig. S7:** **Lidocaine-induced sciatic nerve blockade increases** **incorrect and premature responses in the 3CSRTT following formalin injection.** **A, B** Incorrect rate and premature rate in the 3CSRTT testing of saline / formalin group and lidocaine / formalin group over the 60-min testing period. Data points were displayed in 5-min time bins (n = 7 per group). Data are mean ± SEM. **p* < 0.05. Two-way analysis of variance followed by the Sidak’s multiple comparisons test in **A and B**. 3CSRTT, three-choice serial reaction time task.

**
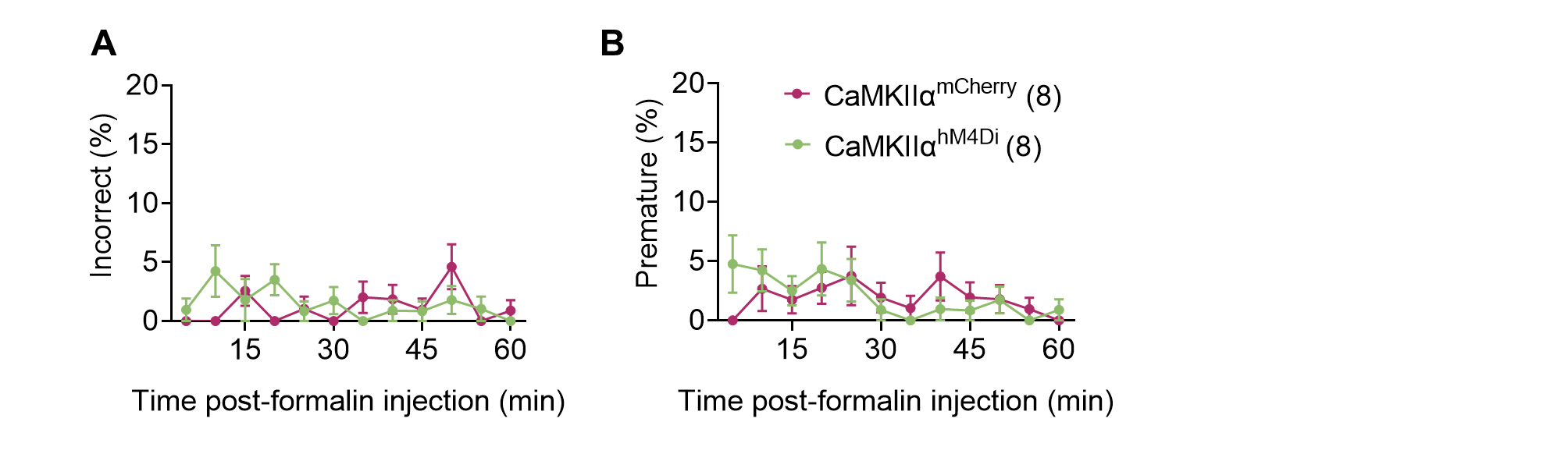
**

**Fig. S8:** **Chemogenetic inhibition of LPBN CaMKIIα neurons has no effect on incorrect and premature in the 3CSRTT following formalin injection. A, B** Incorrect and premature over the 60-min testing period in CaMKIIα^mCherry^ and CaMKIIα^hM4Di^ mice (n = 8 per group). Data points are displayed in 5-min time bins. Data are mean ± SEM. **p* < 0.05. Two-way analysis of variance followed by the Sidak’s multiple comparisons test in **A and B**. 3CSRTT, three-choice serial reaction time task; CaMKIIα, Ca^2+^/calmodulin-dependent protein kinase IIα.

**
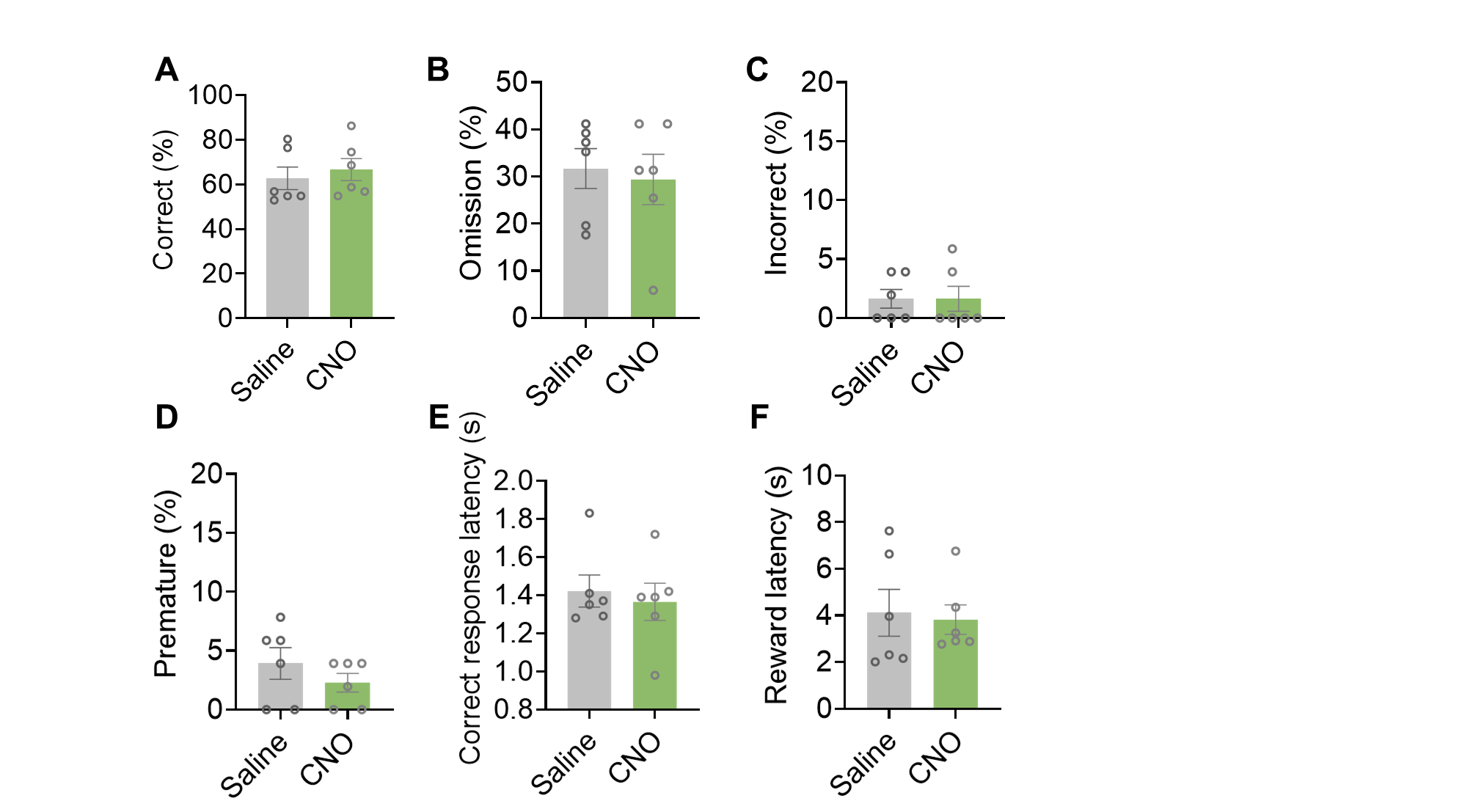
**

**Fig. S9:** **Chemogenetic inhibition of LPBN CaMKIIα neurons has no effect on 3CSRTT performance in the normal state. A-F** Effects of saline or CNO injection on correct rate, omission rate, incorrect rate, premature rate, correct response latency and reward latency in the 3CSRTT in CaMKIIα^hM4Di^ mice (n = 6). Data are mean ± SEM. Two-tailed paired t test in **A-F**. 3CSRTT, three-choice serial reaction time task; CaMKIIα, Ca^2+^/calmodulin-dependent protein kinase IIα.

**
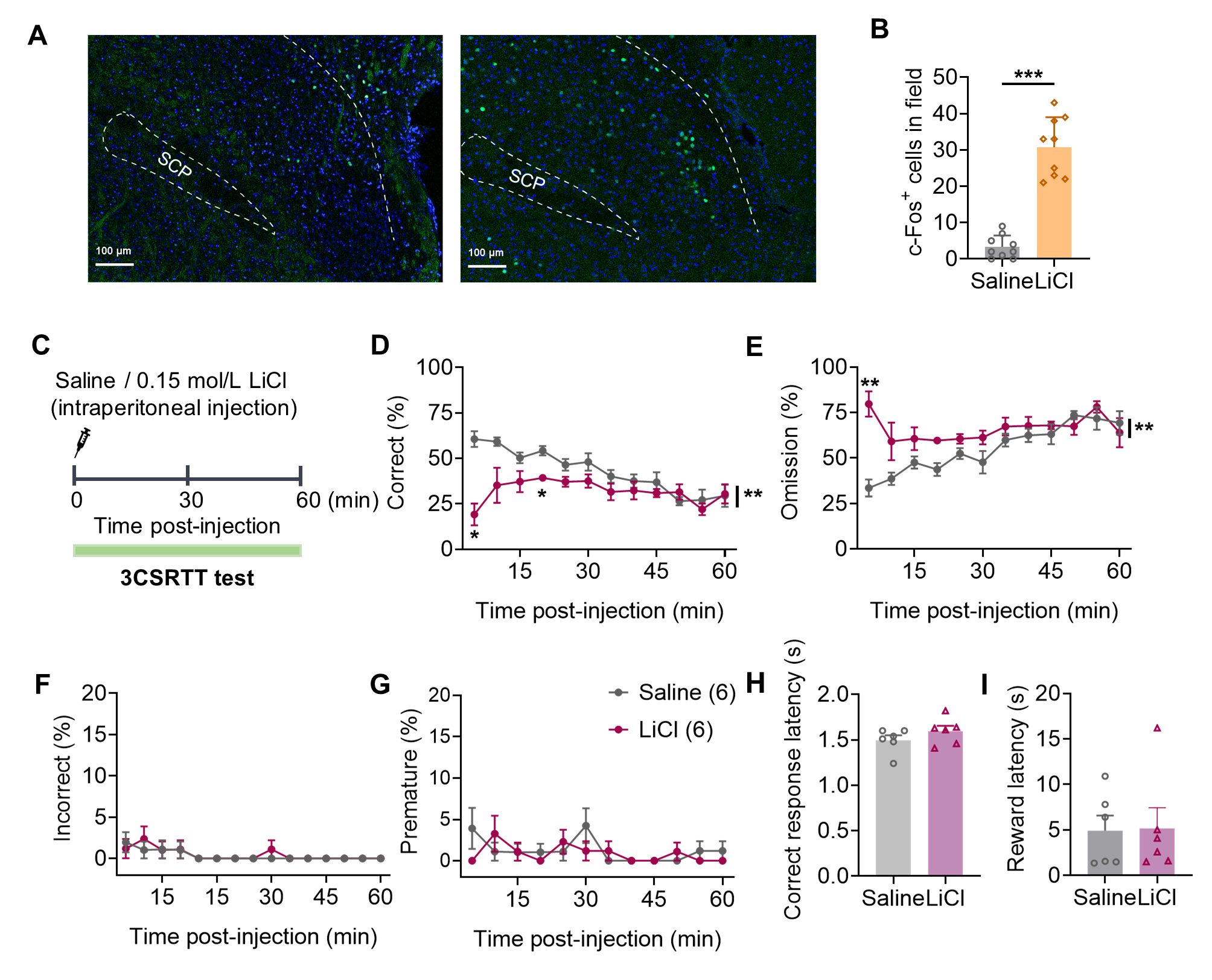
**

**Fig. S10:** **Intraperitoneal injection of lithium chloride (LiCl) impairs 3CSRTT performance. A** Representative images and graphs depicting quantification of c-Fos^+^ neurons **(B)** in the LPBN 1.5 h after saline or LiCl injection (n = 9 sections from 3 mice per group). **C** Experimental design. 3CSRTT was performed immediately after saline or LiCl injection for 60 min. **D-G** Effects of saline or LiCl injection on correct, omission, incorrect, and premature responses in the 3CSRTT during the 60-min testing period. Data points are displayed in 5-min time bins. **H, I** Effects of saline or LiCl injection on reward latency and correct response latency in the 3CSRTT (n = 6 per group). Data are mean ± SEM. Two-way analysis of variance followed by the Sidak’s multiple comparisons test in **D-G**. Two-tailed unpaired t test in **B, H and I**. 3CSRTT, three-choice serial reaction time task; CaMKIIα, Ca^2+^/calmodulin-dependent protein kinase IIα; SCP, superior cerebellar peduncle.
